# Diet Prevents Social Stress-Induced Maladaptive Neurobehavioural and Gut Microbiota Changes in a Histamine-Dependent Manner

**DOI:** 10.3390/ijms23020862

**Published:** 2022-01-13

**Authors:** Alessia Costa, Barbara Rani, Thomaz F. S. Bastiaanssen, Francesco Bonfiglio, Eoin Gunnigle, Gustavo Provensi, Moira Rossitto, Marcus Boehme, Conall Strain, Clara S. Martínez, Patrizio Blandina, John F. Cryan, Sophie Layé, Renato Corradetti, Maria Beatrice Passani

**Affiliations:** 1 Dipartimento di Scienze della Salute, Universitá di Firenze, Viale Pieraccini 6, 50139 Firenze, Italy; alessia.costa@unifi.it (A.C.); barbara.rani@unifi.it (B.R.); 2 APC Microbiome Ireland, University College Cork, T12 YT20 Cork, Ireland; thomaz.bastiaanssen@ucc.ie (T.F.S.B.); eoin.gunnigle@ucc.ie (E.G.); marcus.boehme@outlook.com (M.B.); conall.strain@teagasc.ie (C.S.); clara.martinez@teagasc.ie (C.S.M.); J.Cryan@ucc.ie (J.F.C.); 3 Department of Anatomy and Neuroscience, University College Cork, T12 YT20 Cork, Ireland; 4 Dipartimento di Neuroscienze, Psicologia, Area del Farmaco e Salute del Bambino (NEUROFARBA), Universitá di Firenze, Viale Pieraccini 6, 50139 Firenze, Italy; francescobonfi90@gmail.com (F.B.); gustavo.provensi@unifi.it (G.P.); patrizio.blandina@unifi.it (P.B.); 5 Laboratoire NutriNeuro, UMR INRAE, Bordeaux INP, Université de Bordeaux, 146 Rue Léo Saignat, 33076 Bordeaux, France; moira.rossitto@inrae.fr (M.R.); sophie.laye@inrae.fr (S.L.)

**Keywords:** *Hdc null* mice, n-3 LC-PUFA, long-term potentiation, oxylipins, social avoidance

## Abstract

Exposure to repeated social stress may cause maladaptive emotional reactions that can be reduced by healthy nutritional supplementation. Histaminergic neurotransmission has a central role in orchestrating specific behavioural responses depending on the homeostatic state of a subject, but it remains to be established if it participates in the protective effects against the insults of chronic stress afforded by a healthy diet. By using C57BL/6J male mice that do not synthesize histamine (*Hdc^−/−^*) and their wild type (*Hdc*^+/+^) congeners we evaluated if the histaminergic system participates in the protective action of a diet enriched with polyunsaturated fatty acids and vitamin A on the deleterious effect of chronic stress. Behavioural tests across domains relevant to cognition and anxiety were performed. Hippocampal synaptic plasticity, cytokine expression, hippocampal fatty acids, oxylipins and microbiota composition were also assessed. Chronic stress induced social avoidance, poor recognition memory, affected hippocampal long-term potentiation, changed the microbiota profile, brain cytokines, fatty acid and oxylipins composition of both *Hdc*^−/−^ and *Hdc*^+/+^ mice. Dietary enrichment counteracted stress-induced deficits only in *Hdc*^+/+^ mice as histamine deficiency prevented almost all the diet-related beneficial effects. *Interpretation*: Our results reveal a previously unexplored and novel role for brain histamine as a mediator of many favorable effects of the enriched diet. These data present long-reaching perspectives in the field of nutritional neuropsychopharmacology.

## 1. Introduction

Repeated exposure to stressful stimuli is a well-known risk factor for the development of anxiety and mood disorders. Chronic stressors are defined as persistent events which require an individual to make adaptations over an extended period. The experienced emotional, behavioural, and physiological toll can predispose an individual to a greater risk of developing mental disorders and physical illness [1]. Indeed, the majority of human psychopathologies are of a social nature and are associated with cognitive decline [2]. Chronic social stress is a prominent contributor to mood disorder prevalence and suicide attempts in victims of bullying [3]. Furthermore, stress-induced neuropsychiatric disturbances have been associated with altered neuroplasticity, as well as microglia activation and neuroinflammatory signalling (reviewed in [4]).

Chronic social defeat stress (CSDS) is a murine stress model that recapitulates crucial behavioural, neuroendocrine and immunological modifications observed in humans exposed to social stress [5]. Mice subjected to CSDS display a long-lasting reduction in social interaction [6], altered organisation of behavioural sequences and memory deficits [7], neurochemical and neurotransmitter changes [8] as well as inflammatory responses throughout the brain [9]. Consequently, several homeostatic processes are chronically affected [10], and some of these alterations are reversed by chronic treatment with antidepressants [11]. These are not always effective, and their use is inevitably associated with side effects. Hence, there is a strong necessity to explore additional approaches for improving clinical outcomes and find novel preventive or corrective strategies to decrease the incidence of cognitive disorders.

We recently provided evidence that micronutrients such eicosapentaenoic (EPA), docosahexaenoic (DHA), long chain polyunsaturated fatty acids (n-3 LC-PUFAs) and vitamin A prevent the cognitive deficits induced by social instability stress during adolescence in rats: rats fed a diet enriched with n-3 LC-PUFA+VitA exhibited improved performance in both emotional and reference memory tests, and hippocampal BDNF expression indistinguishable from non-stressed rats [12]. Importantly, the stress-induced effects on the gut microbiome were also ameliorated by the diet, though it was unclear if these findings were mechanistically linked. These dietary lipids play an indispensable role in CNS development, anti-inflammatory processes [13], they possess neuroprotective properties and affect neuronal plasticity [14]. Additionally, vitamin A plays a key role in regulating synaptic plasticity, learning and memory through its active metabolite, retinoic acid [15].

Here, we address the questions if the brain histaminergic system and peripheral mechanisms participate in the protective effects of the n-3 LC-PUFA+VitA enriched diet towards chronic stress-induced disturbances. In this regard, we showed that the brain histaminergic system serves as a relay station integrating peripheral signals, such as the satiety factors oleoylethanolamide (OEA; [16] or glucacon-like peptide 1 (GLP-1; [17]. It also plays a crucial role in central functions, such as fear memory consolidation [18] and retrieval [19], to influence the homeostatic or emotional value of different experiences. Hypothalamic histaminergic neurons are profoundly affected by acute stressors, such as restraint stress, insulin-induced hypoglycemia or foot-shock [20]. Also, the expression of histaminergic receptors in different rat brain areas is highly sensitive to both acute and prolonged stress exposure [21]. Histaminergic neurons are clustered in the posterior hypothalamus and despite their diffuse projections throughout the brain, they appear to display selective activity during the unfolding of specific behaviours [22]. This prompts the question whether the histaminergic system plays a role in the protective action of a n-3 LC-PUFA+VitA enriched diet against chronic stress. To assess the consequences of chronic stress, we subjected modified mice that cannot synthesize histamine (histidine decarboxylase knockout (*Hdc*^−/−^) and *Hdc*^+/+^ mice to a comprehensive battery of behavioural tests ranging from recognition memory to social interaction and anxiety-like behaviour. As repeated social defeat profoundly affects hippocampal morphology and neurophysiology [23], we also examined the induction of long-term potentiation (LTP) in the dorsal hippocampal CA1, a region involved in temporal pattern association and intermediate term memory [24]. There is vast literature implicating inflammatory mechanisms as a consequence of stressful experiences that may precipitate depression and boost proinflammatory cytokine production [25,26]. n-3 LC-PUFAs exert anti-inflammatory properties in part through the synthesis of oxylipins, specialised pro-resolving mediators [27,28,29]. We therefore analysed hippocampal PUFA composition, the production of cytokines and oxylipins and their metabolic pathways, as oxylipins have gained relevance in the regulation of stress-induced neuroinflammation, characterised by brain monocyte entry and pro-inflammatory cytokine production [13,30,31]. As there is bidirectional communication between the state of the microbiota and stress mechanisms [32,33], we also investigated the impact of CSDS and our dietetic intervention on the gut microbiota composition of both genotypes. The gut microbiota plays a crucial role in shaping host homeostasis, regulating brain function and behaviour, and producing neurotransmitters and neuroactive compounds [34]. These compositional changes could interfere with the metabolic activity of the gut microbiota, and may, in turn, impact gut–brain axis communication.

## 2. Materials and Methods

### 2.1. Animals and Ethical Approval

C57BL/6 histidine decarboxylase null (*Hdc^−/−^*) and wild type (*Hdc*^+/+^) male mice were grown in the Centro Stabulazione Animali di Laboratorio (CeSAL), Università di Firenze in a humidity and temperature-controlled room (22–24 °C), were allowed free access to food and water, and kept on a 12-h light/dark cycle (lights start at 7:00 a.m.). *Hdc*^−/−^ mice genotype was determined by PCR analysis as described in Provensi et al. [12]. At postnatal (PND) day 21, mice were weaned and randomly allocated to receive control diet (Mucedola s.r.l., Milan, Italy;) or a diet enriched in n-3 LC-PUFA and vitamin A (Sniff, Munich, Germany). Diets were matched for macronutrient content as shown in Table S1. Food was changed and weighed every day. Nine to 13-week-old male CD1 retired breeders (Charles River, Italy) were screened for aggressive behaviour and used for the social defeat stress protocol according to Golden et al. [35]. All experiments were performed in accordance with the EEC recommendations for the care and use of laboratory animals (2010/63/EU) and approved by the Animal Care Committee of the University of Florence and Italian Ministry of Health (authorization n. 114-2017PR). Animal experiments were carried out in accordance with the Ethical Policy of the Universitá di Firenze which complies with the Guide for the Care and Use of Laboratory Animals of the Council Directive of the European Community (2010/63/EU) and the Italian Decreto Legislativo 26 (13 March 2014). Every effort was made to minimize animal suffering and to reduce the number of animals used. All animals were weighed, and food consumption was calculated daily.

### 2.2. Chronic Social Defeat Stress

C57BL/6 mice were singly housed for 4 days prior to undergoing social defeat stress. CD1 male mice were used as resident aggressors for the social defeat stress and were singly housed prior to the experiments. Aggressive CD-1 mice, as defined by demonstrating at least one successful act of aggression during two consecutive days toward another male CD-1 intruder mouse, were selected for use during the social defeat. A random group of PND56 *Hdc*^+/+^ and of *Hdc*^−/−^ mice that had been fed with the control diet (SCD) and a group of mice of either genotype that had been fed with the enriched diet (SED) were subjected to the CSDS protocol as reported in Rani et al. [7]. Non-stressed control mice (NS) were left undisturbed in their own home cage with other NS mice, as group-housed siblings have been regarded as a valid control group in social stress studies [36]. Details are provided in the Supplementary Material.

### 2.3. Social Interaction Test

The test is used in rodents to assess sociability and interest in social novelty [37]. Following repeated exposure to aggressive CD1 mice, C57BL/6 experimental animals display marked social avoidance. Mice were subjected to the test 24 h after the last defeat session adopting a protocol from Golden et al. [35]. Briefly, mice were habituated to the arena (41 × 32 × 40 cm) that contained an empty wire-mesh enclosure (7.5 cm length, 9.5 cm width). Movements were recorded for 2.5 min to determine baseline exploratory behaviour and locomotion in the absence of the social target within the enclosure (T1). In the second session, conditions were identical except that the wire-mesh contained a CD1 aggressive mouse defined as the ‘target’. During the test (presence of the social target, T2) the experimental C57BL/6 mouse was reintroduced to the arena containing an unfamiliar CD1 mouse within the wire cage and the time spent by the experimental mouse in the interaction was measured. Social interaction (SI) behaviour was estimated as a ratio dividing the time spent in the interaction zone (virtual area around the cage) during T2 by the time spent in the interaction zone during T1. After the social interaction tests, mice were allocated to different experimental groups, and subjected to cognitive, neurochemical or electrophysiological testing.

### 2.4. Novel Object Recognition Test

The NOR paradigm consisted of habituation, training (T1) and test (T2). Twenty-four hours after a 10-min habituation period, mice were placed in the test arena containing two identical plastic objects (cubes or pyramids). The time spent actively exploring the objects during the 5 min T1 was recorded by an experienced observer unaware of the treatments. Exploration was defined as sniffing or touching the objects with the nose and/or forepaws. Sitting on or turning around the objects was not considered exploratory behaviour. T2 was performed 1 h after T1, during which, each mouse was again placed in the test arena for 5 min in the presence of one of the familiar objects and a novel object, and the time spent exploring either object was again recorded. The position of the objects (left/right) was randomized to prevent bias from order or place preference.

### 2.5. Novel Object Location Test

The novel object location (NOL) paradigm consisted of habituation, training (T1) and test (T2). Twenty-four hours after a 10-min habituation period, mice were placed in the test arena containing two plastic objects such as cubes or pyramids. The time spent actively exploring the objects during the 5 min of T1 was recorded by an experienced observer unaware of the treatments. Exploration was defined as sniffing or touching the objects with the nose and/or forepaws. Sitting on or turning around the objects was not considered as exploratory behaviour. T2 was performed 1 h after T1, during which, each mouse was again placed in the test arena for 5 min in the presence of the same two objects, but one of them displaced in a different position within the arena. Time spent exploring either object was recorded.

### 2.6. Open Field Test

Anxiety-like behaviours and locomotor activity were assessed in the open field consisting of a 60 × 70 × 30 cm square arena. A virtual zone (20 × 23 cm) was delimited in the center of the arena. Mice were placed facing the center of the arena and allowed to freely explore for 10 min. After each observation, the arena was cleaned with 30% ethyl alcohol in water to remove possible scent cues left by the animal. The time spent at the center and periphery of the open field and total distance travelled were recorded using the video tracking system AnyMaze and analyzed using Smart 2.5 software.

### 2.7. Western Blot Analysis

For neurochemical determinations, mice were sacrificed 24 h after the end of behavioural evaluations. Brains were dissected out on ice and hippocampi immediately isolated. Details of the procedure are provided in the Supplementary Material.

### 2.8. Long-Term Potentiation

*Hippocampal slices preparation*: Hippocampal slices were prepared from 10-week-old experimental mice following the social interaction test (SIT) as previously described [38,39,40]. Mice were anaesthetized with isofluorane and decapitated with a scissor cut. The hippocampi were rapidly removed and placed in ice-cold artificial cerebrospinal fluid (ACSF), which contained the following (in mM): NaCl, 124; KCl, 2.75; NaH_2_PO_4_, 1.25; NaHCO_3_, 26; MgSO_4_, 1.3; CaCl_2_, 2.5; D-glucose 10. The solution was bubbled with a 95% O_2_/5% CO_2_ gas mixture (pH 7.4). After discarding approximately 2 mm of the dorsal hippocampal pole, six transversal slices of 400 µm nominal thickness were cut with a McIlwain tissue chopper (Gomshall, UK) and kept for at least 1 h at room temperature until recording. Typically, two out of the six (dorsal–central) slices were used for LTP experiments. *Electrophysiological recordings:* Before transferring the slice to the recording chamber, the CA1 area was disconnected from the CA3 area by a surgical cut. The slice was then placed on a nylon mesh, completely submerged in a recording chamber and continuously superfused (2 mL min-1) with oxygenated ACSF at 32–33 °C. Slices were incubated for 15 min in the recording chamber before initiating electrical stimulation. Synaptic responses of CA1 pyramidal neurons apical dendrites were elicited by stimulation of the Schaffer collateral/commissural pathway. Stimulation pulses (80 µs duration; 30 s interpulse interval), triggered by a PC controlled by WinLTP software [41] were delivered by a stimulus isolation unit (DS2, Digitimer, Welwyn Garden City, UK) through a concentric bipolar Pt-Ir electrode (125 µm/Rnd/25 µm; FHC, Bowdoin, ME, USA). Evoked potentials were recorded with glass electrodes filled with 150 mM NaCl (2–10 MΩ resistance) placed in the distal third of the stratum radiatum to record field excitatory postsynaptic potentials (fEPSP). The distance between the stimulating and recording electrodes was 300–400 µm. Recorded potentials were amplified with a Neurolog NL 104 amplifier (Digitimer), digitized with the sampling rate of 50 kHz (Digidata 1322A, Molecular Devices, Foster City, CA, USA) and stored in a PC for offline analysis using WinLTP software. At the beginning of each experiment, a stimulus–response curve (SRC), obtained by gradually increasing stimulus intensity, was recorded. The fEPSP was measured as the slope of the initial falling phase of the response recorded in the stratum radiatum following the afferent volley and measured by linear regression in the region between 30 and 70% of the fEPSP amplitude. The stimulus intensity of test pulses was set to evoke a fEPSP that had an initial slope of ~40% (range 35–45%) of the maximum obtained in the SRC, and stimulus intensity was held constant throughout the remainder of the experiment. *LTP protocol:* At least 20 min of stable responses were used to generate the baseline values, before inducing LTP. LTP was induced by theta-burst stimulation consisting of a single train of 5 bursts of 5 stimuli (100 Hz intra-burst frequency, 5 Hz burst frequency; called TB5). TB5 was chosen among the possible patterns of theta rhythm-based stimulations because it produces LTP of intermediate magnitude, thus allowing for detection of modulatory effects in both inhibitory and facilitatory directions. Responses to test stimuli were followed for 60 min after LTP induction. Steady-state values of potentiation were obtained by averaging the values of the 11 consecutive responses recorded over the 5-min period between 55 and 60 min after TB5 stimulation.

### 2.9. Real-Time PCR of Gene Expression in the Hippocampus

The hippocampi were rapidly removed and stored at −80°C. RNA was extracted using TRIzol reagent (Invitrogen, Life Technologies™, Saint-Aubin, France). RNA concentrations were determined using a Nanodrop ND-1000 (Labtech). Using OligodT and random primers (Invitrogen), cDNA was synthesized with SuperScript IV Reverse Transcriptase (Invitrogen, Life Technologies™, Saint-Aubin, France). Briefly, 1 µg of total RNA mixed with RNasin (Invitrogen, Life Technologies™, Saint-Aubin, France) and DNase (Invitrogen, Life Technologies™, Saint-Aubin, France) was incubated at 37 °C. OligodT and random primers were added for incubation at 65 °C. Then, the SuperScript IV mix was added, and the mixtures were incubated at 23 °C for 10 min, followed by 50 °C for 10 min and 80 °C for 10 min. To measure fatty acid metabolic enzymes, quantitative PCR was performed using the Applied Biosystems (Foster, CA, USA) assay-on demand gene expression protocol as previously described [42]. Details are provided in the Supplementary Materials.

### 2.10. Oxylipins Analysis

All tissues were snap-frozen in liquid nitrogen immediately after collection and stored at −80 °C until extraction. For extraction, each frozen tissue was crushed with a FastPrep^®^-24 Instrument (MP Biomedical, Santa Ana, CA, USA) in 500 µL of HBSS (Invitrogen). After 2 crush cycles (6.5 m/s, 30 s), 10 µL were withdrawn for protein quantification. 300 µL of cold methanol and 5 µL of internal standard (Deuterium labelled compounds) were added to homogenates. After centrifugation at 2000 *g* for 15 min at 4 °C, supernatants were transferred into 2 mL 96-well deep plates and diluted in H_2_O to 2 mL. Samples were then submitted to solid phase extraction (SPE) using an OASIS HLB 96-well plate (30 mg/well, Waters) pre-treated with MeOH (1 mL) and equilibrated with 10% MeOH (1 mL). After sample application, the extraction plate was washed with 10% MeOH (1 mL). After drying under aspiration, lipid mediators were eluted with 1 mL of MeOH. Prior to LC-MS/MS analysis, samples were evaporated under nitrogen gas and reconstituted in 10 µL of MeOH.

LC-MS/MS analyses of eicosanoids were performed as previously described [30]. Briefly, lipid mediators were separated on a ZorBAX SB-C18 column (2.1 mm, 100 mm, 1.8 µm) (Agilent Technologies, Santa Clara, CA, USA) using Agilent 1290 Infinity HPLC system (Technologies) coupled to an ESI-triple quadruple G6460 mass spectrometer (Agilent Technologies, Santa Clara, CA, USA). Data were acquired in the Multiple Reaction Monitoring (MRM) mode with optimized conditions (ion optics and collision energy). Peak detection, integration and quantitative analysis were done using Mass Hunter Quantitative analysis software (Agilent Technologies, Santa Clara, CA, USA) based on calibration lines built with commercially available eicosanoids standards (Cayman Chemicals, Ann Arbor, MI, USA).

### 2.11. Fatty Acid Analysis

Total lipids were extracted according to the method developed by Folch et al. [43]. They were submitted to fatty acid methylation using 7% boron trifluoride in methanol according to Morrison and Smith [44]. Fatty acid methyl esters (FAMEs) were analyzed using gas chromatography on a Hewlett Packard Model 5890 gas chromatograph (Palo Alto, CA, USA) using a CPSIL-88 column (100 m × 0.25 mm internal diameter; film thickness, 0.20 μm; Varian, Les Ulis, France). Hydrogen was used as a carrier gas (inlet pressure, 210 kPa). The oven temperature was held at 60 °C for 5 min, increased to 165 °C at 15 °C/min and held for 1 min, and then to 225 °C at 2 °C/min and finally held at 225 °C for 17 min. The injector and the detector were maintained at 250 °C and 280 °C, respectively. FAMEs were identified by comparison with commercial and synthetic standards. The data were computed using the Galaxie software (Varian). The proportion of each fatty acid was expressed as a percentage of total fatty acids to allow the comparison of lipid composition in different mouse strains and different conditions as previously described [30].

### 2.12. Microbiome Analysis

Fecal pellets were collected at T0 before the initiation of the social stress protocol and T1, the last day of the stressful procedure, in sterile conditions and stored at −80 °C until extraction of nucleic acids. DNA was extracted from 250 mg fecal samples using a previously described modified protocol, which combined the repeat bead beating method with the QIAamp Fast DNA Stool Mini Kit (Qiagen, Germany) [45]. DNA was quantified using the QubitTM 3.0 Fluorometer (Bio-Sciences Dublin, Ireland or Life Technologies or Thermo Fisher Scientific) and the Qubit^®^ dsDNA HS Assay Kit (Bio-Sciences Dublin, Ireland or Life Technologies or Thermo Fisher Scientific), along with being run on a 1.5% agarose gel to check the DNA quality. Extracted DNA was stored at −20 °C until prepared for 16s rRNA sequencing. The V3-V4 hypervariable region of the 16S rRNA gene was amplified from the DNA extracts and prepared for sequencing using the Illumina 16S Metagenomic Sequencing Library Protocol (Amplicon). A detailed description of the 16S rRNA sequencing protocol can be found in the Supplementary Methods. *Microbiome Pre-processing:* Three hundred base paired-end reads were pre-filtered based on a quality score threshold of >28, trimmed and filtered for quality and chimaeras using the DADA2 library [46] in R (version 3.6.3). Only samples with >10,000 reads after quality control steps were used in the analysis. Taxonomy was assigned using the DADA2 package that included the SILVA SSURef database release v132. Parameters as recommended in the DADA2 manual were adhered to unless mentioned otherwise. ASVs were aggregated at genus level; those that were unknown at the genus level were not considered in the downstream analysis.

### 2.13. Quantification and Statistical Analysis

Data from behavioural and neurochemical experiments were analyzed using Prism 6 software (GraphPad Software, San Diego, CA, USA). The data presented in the graphs are expressed as mean ± S.E.M. Statistical significance was determined using a two-way ANOVA followed by Bonferroni’s multiple comparison post hoc tests. The level of significance was set to *p* < 0.05. For statistical comparison of changes in the LTP experiments, the steady-state values were computed by averaging 11 consecutive responses obtained over a 5 min period immediately before the theta burst stimulation (baseline value) and at 55–60 min after TB5. Typically, more than one slice was used per mouse and the results of all determinations per genotype and treatment were analyzed to account for the overall variability of LTP responses in the different genotypes. Mean values from replicates in the same animal were used for the genotype vs condition statistical analysis reported in results. Unless otherwise stated, numbers represent experiments carried out in slices taken from different animals. Results of statistical analysis are provided in Tables S4 and S5. Further data-handling was undertaken in R (version 3.6.3) using RStudio GUI (version 1.1.453).

### 2.14. Microbiome Statistical Analysis

In all microbiome analysis except for alpha diversity, taxa with a prevalence of <5% of samples at the genus level were excluded from the analysis as ratios are invariant to the subsetting and this study employs compositional data analysis techniques [47,48]. Principal component analysis was performed on centred log-ratio transformed (clr) values using the ALDEx2 library. The number of permutations was set to 1000. Beta diversity was computed in terms of Aitchison distance, or Euclidean distance between CLR-transformed data. Alpha diversity was computed using the iNEXT library [49]. Piphillin was used for functional inference from 16S rRNA gene sequences in the form of KEGG orthologues [50]. Gut-Brain Modules (GBMs) and Gut-Metabolic Modules (GMMs) were calculated using the R version of the Gomixer tool [51]. Stacked bar plots were generated by normalizing counts to 1, generating proportions. Genera that were never detected at a 2% relative abundance or higher were aggregated and defined as rare taxa for the purposes of the stacked bar plots. In cases where more than two factors were considered, the lme4 library in R was used to fit the linear mixed effect models, with animal ID as a random effect if applicable [52]. Differential abundance of both microbes and functional modules were calculated using implementation of the ALDEx2 library. A *p*-value of <0.05 was deemed significant in all cases. To correct for multiple testing in tests involving microbiota or functional modules, the Benjamini–Hochberg (BH) post hoc was performed with a q-value of 0.1 used as a cut-off. Custom R scripts and functions are available at https://github.com/thomazbastiaanssen/Tjazi (accessed on 12 December 2021). All experiments were carried out by personnel blind to group and outcome assessment.

## 3. Results

Overall, non-stressed *Hdc*^+/+^ and *Hdc*^−/−^ mice did not differ significantly in their behavioural, neurochemical and electrophysiological responses. Furthermore, the two genotypes responded similarly to CSDS. However, the association stress and diet revealed major differences between genotypes.

### 3.1. Effect of Stress and the Enriched Diet on Body Weight and Food Consumption

**Figure 1:**
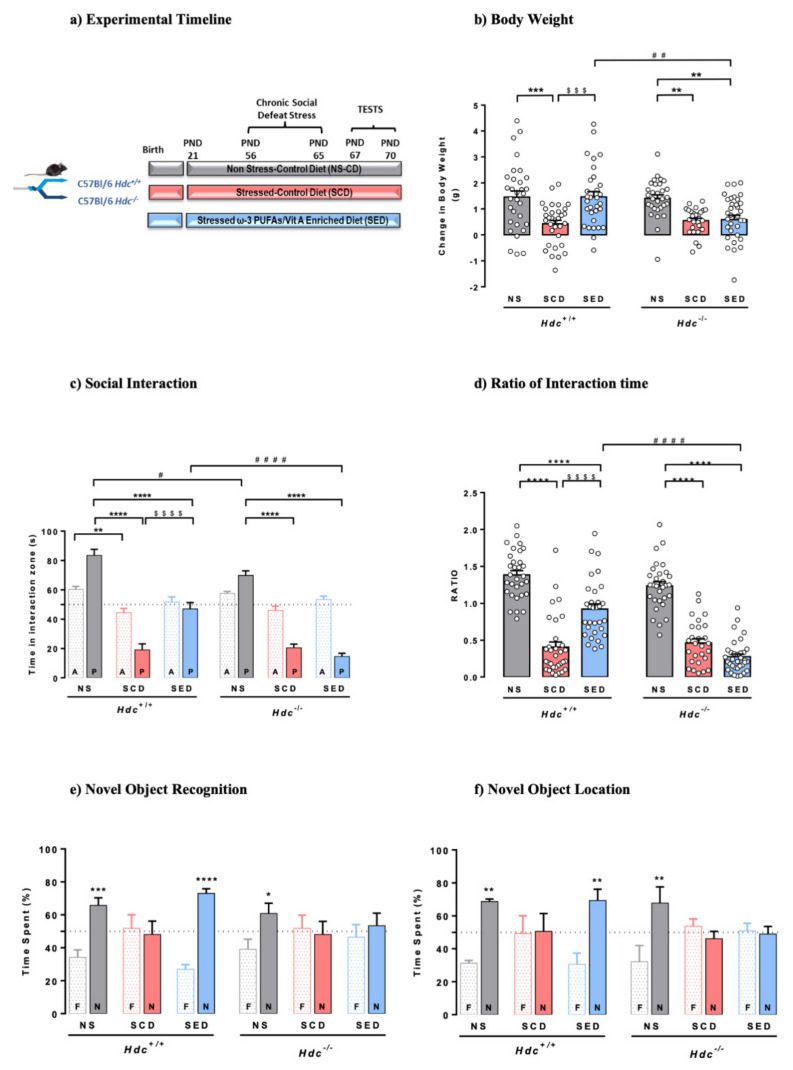
(**a**) Timeline for the chronic social defeat stress protocol and diet supplementation. Mice were randomly assigned to three experimental groups per genotype. (**b**) Effects of stress and n-3 LC-PUFA+VitA enriched diet on weight gain. Data are expressed as means ± S.E.M. of 27–34 mice per experimental group. *** *p* < 0.001; ** *p* < 0.01 vs. respective NS mice; ^$$$^ *p* < 0.001 vs. SCD mice; ^##^ *p* < 0.01 vs. same treatment of different genotype. NS = non-stressed; SCD = stressed, control diet; SED = stressed, enriched diet; (**c**,**d**) Effect of stress and the enriched diet on social-avoidance behaviour induced by stress expressed as time in the interaction zone (**c**) and social interaction ratio (**d**). P, target present; A, target absent. Data are represented as means ± S.E.M. of 27–34 mice per experimental group. **** *p* < 0.0001; ** *p* < 0.01 vs. respective NS mice; ^$$$$^ *p* < 0.0001 vs. respective SCD mice; ^####^ *p* < 0.001, ^#^ *p* < 0.05 vs. same treatment of different genotype. (**e**,**f**) Effect of the enriched diet on stress-induced cognitive impairment in the novel object recognition test (**e**) and novel location recognition (**f**) Data are represented as means ± S.E.M. of 5–9 mice per experimental group in the NOR and mean ± S.E.M. of 5-6 mice per experimental group in the NOL. N, novel object; F, familiar object. **** *p* < 0.0001; *** *p* < 0.001, ** *p* < 0.01, * *p* < 0.05 vs. familiar object; NS = non-stressed; SCD = stressed, control diet; SED = stressed, enriched diet.

Figure 1a shows the timeline of the experimental protocol. The procedure and diet had significant consequences on mouse body weight. At the end of the stressful procedure, stressed mice of both genotypes fed with the control diet (SCD) had gained less weight than non-stressed (NS) mice (Figure 1b). These results are in line with ours and others’ social stressful protocols [12,53]. Stressed *Hdc*^+/+^ mice fed the enriched diet (SED) gained significantly more weight than SCD mice (*p* < 0.001), however, the enriched diet was ineffective in stressed *Hdc*^−/−^ mice. Despite the significant differences in final body weight, mice of either genotype eat comparable amounts of food regardless of diet or stress (Figure S1a). In this regard, we previously showed a similar trend in rats subjected to the social instability stress [12]. This is not surprising as the physiological and behavioural interactions that link stress, food intake and emotional state are extremely complex [54].

### 3.2. The Enriched Diet Prevents the Development of Social Avoidance of Hdc^+/+^ but Not of Hdc^−/−^ Mice

CSDS consists of agonistic social confrontations between an aggressive and an experimental mouse, and assessment of social avoidance in the subordinate animals [35]. Mice that were repeatedly subjected to bouts of social defeat by a larger and aggressive CD1 mouse developed a stress-susceptible behaviour, here expressed as less time spent in the interaction zone with a novel animal present (Figure 1c), and a lower social interaction ratio (Figure 1d) compared with NS mice of either genotype, which displayed normal sociability. The enriched diet prevented the social behaviour of *Hdc*^+/+^ mice, as they spent significantly more time in the interaction zone then SCD congeners but was completely ineffective in SED *Hdc*^−/−^ mice. The social interaction test and the ratio of interaction time is used to discriminate between resilient and susceptible mice [55,56]. Indeed, considering a cut off value = 1 [55], most SCD mice of either genotype were “susceptible”. In the case of SED *Hdc*^+/+^ mice, it is virtually impossible to discriminate the beneficial effect of the diet from resiliency, as the dietary intervention started well before the stress protocol (Figure 1a). Nonetheless, *Hdc*^−/−^ SED mice are all under the ratio point of resilience and appear to be susceptible to stress. These results suggest pro-resilient effects of the supplemented diet in *Hdc*^+/+^ mice which is not observe in histamine-depleted mice.

### 3.3. Locomotor Activity and Anxiety-like Related Behaviours of Hdc^+/+^ and Hdc^−/−^ Mice

Mice performance in the social interaction test was not due to locomotor impairment. Locomotion measured as the distance travelled and the time spent moving in both the centre and periphery of the arena, were not different among the experimental groups of either genotype. Stress, diet and genotype did not affect the number of entries in the central zone (Figure S1b), the distance travelled (Figure S1c), the time spent moving (except for SCD *Hdc*^+/+^ mice; Figure S1d), the time spent in the centre or periphery of the open field (Figure S1e). Hence, within the arena, mice of both genotypes did not show overt signs of anxiety-like behaviour.

### 3.4. The Enriched Diet Prevents Stress-Induced Memory Impairment of Hdc^+/+^, but Not That of Hdc^−/−^ Mice

CSDS had similar detrimental effects on object recognition memory in both genotypes, as they did not discriminate between the familiar and novel object when tested 1 h after training (Figure 1e). The enriched diet completely prevented the CSDS-induced object discrimination impairment of *Hdc*^+/+^ mice. However, the enriched diet did not prevent the memory impairment of *Hdc*^−/−^ mice, as these did not discriminate between the familiar and novel object (Figure 1e). To further assess cognitive functioning, memory recall was tested using a spatial version of the object recognition, the novel location recognition paradigm (Figure 1f). A two-way ANOVA analysis showed significant interactions between variables. SCD mice of either genotype behaved in a similar way as in the novel object recognition test (Figure 1f), and the enriched diet prevented the deleterious effect of stress in *Hdc*^+/+^ mice, whereas it was ineffective in *Hdc*^−/−^ mice.

### 3.5. Effects of the Enriched Diet on Stress-Induced Changes of Hippocampal Synaptic Plasticity

**Figure 2:**
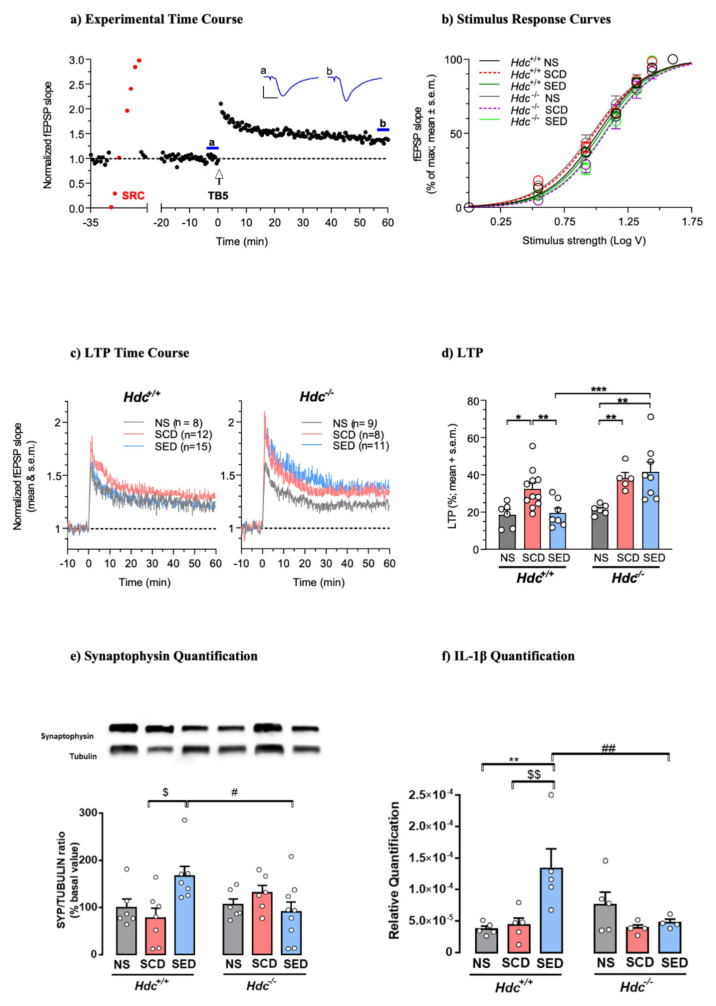
(**a**) Histamine deficiency prevents n-3 PUFA and Vitamin A enriched diet modulation of synaptic plasticity in dorsal hippocampal CA1 region. (**a**) Time-course of a typical experiment. Left: stimulus–response curve (SRC; red points) used to set the stimulus strength to obtain a baseline fEPSP ~35% of maximal response. Right: effect of theta burst stimulation (TB5, arrow). Symbols represent fEPSP slope values normalised to the mean of baseline values measured from 20 to 0 min before TB5. Inset: traces are the average of 7 responses obtained at times indicated by corresponding letters in the time-course (blue lines). Calibration: 1 mV, 5 ms. (**b**) SRCs at baseline were similar across genotypes and treatments. Lines are the best least-squares fit to the logistic equation of average SRC for each experimental group. Symbols represent means ± S.E.M. of responses at selected stimulus intensities (*Hdc^+/+^:* NS, SCD, SED n = 15,28,26 slices; *Hdc*^−/−^: NS, SCD, SED n = 9,9,19 slices; ANOVA of EStim_50_ voltage values obtained in individual SRCs: F_(5, 100)_ = 1.002; *p* = 0.4207). (**c**) Time-course of LTP in slices obtained from *Hdc*^+/+^ and *Hdc*^−/−^ in all experimental conditions. Data are expressed as means ± S.E.M. of fEPSP slope values normalised to the baseline mean from 10 to 0 min before TB5 (delivered at time 0). Numbers (n) in graphs indicate slices tested, including replicates per mouse. (**d**) Summary scatter plot of LTP produced by TB5. Data in columns are expressed as means + S.E.M of the percent change in responses measured 55-60 min after LTP induction with respect to baseline as in (**c**) *Hdc^+/+^:* NS, SCD, SED n = 6,11,7 mice; Hdc^−/−^: NS, SCD, SED n = 6,5,8 mice. Two-way ANOVA: followed by Bonferroni post hoc test * *p* < 0.05, ** *p* < 0.01, *** *p* < 0.001. (**e**,**f**) Effect of stress and diet on hippocampal markers of synaptic plasticity and cytokines. (**e**) Representative immunoblots and densitometric quantification of synaptophysin in hippocampal tissue. Data are expressed as means ± S.E.M. of 6–9 samples per experimental group. (**f**) Densitometric quantification of IL-1β in hippocampal tissue. Data are expressed as mean ± S.E.M of 4–5 samples per experimental group. ** *p* < 0.01, vs respective NS mice; ^$$^ *p* < 0.01, ^$^ *p* < 0.05 vs respective SCD mice; ^##^ *p* < 0.01, ^#^ *p* < 0.05 vs same treatment of different genotype.

The hippocampus is one of the brain structures most involved in regulating stress responses, as previously shown [57,58]. To examine the effect of stress and enriched diet on synaptic plasticity in the two genotypes, we assessed the magnitude of LTP in hippocampal brain slices. Hippocampal LTP was evoked by TB5, one brief train of electrical stimulation which mimics the physiological θ-rhythm and leads to a sustained increase in synaptic transmission efficacy (Figure 2a). This allows the detection of modulatory effects of treatments in both inhibitory and facilitatory directions [59]. Under baseline conditions, the relationship between stimulus strength and response showed similar stimulus voltages to produce 50% of maximal fEPSP responses (EStim50) across genotypes and treatments (Figure 2b) indicating that stress and diet did not significantly affect basal CA1 neurotransmission in either genotype. Induction of LTP produced a significant increase in the fEPSP slope for at least 60 min after TB5 in all groups, although the magnitude differed across treatments in the two genotypes. LTP of SCD mice of both genotypes was significantly greater than that of NS mice. The enriched diet prevented LTP increase in *Hdc*^+/+^ mice, but was ineffective in *Hdc*^−/−^ mice (Figure 2c,d) indicating that the presence of histamine in vivo is required for the diet to re-establish synaptic plasticity to normal levels.

We also investigated the effect of CSDS and diet on hippocampal synaptophysin, a protein implicated in synaptic transmission, in both *Hdc*^+/+^ and *Hdc*^−/−^ mice. Synaptophysin expression in the hippocampus of mice of both genotypes was not significantly affected by CSDS (Figure 2e), which is in agreement with previous observations using different stressful protocols [60]. The enriched diet increased synaptophysin expression in the hippocampus of SED *Hdc*^+/+^ mice in accordance with the observations by Venna et al. [61]. However, the enriched diet did not affect synaptophysin expression in the hippocampus of stressed *Hdc*^−/−^ mice (Figure 2e).

### 3.6. Diet Affects the Expression of Cytokines in Hdc^+/+^ Mice Hippocampus

Cytokines have been described as homeostatic factors in hippocampal physiology [62]. In the hippocampus, IL-1β production was not affected by stress in either genotype; however, the enriched diet increased the IL-1β level in the hippocampus of *Hdc*^+/+^ mice but was ineffective in null mice (Figure 2f). IL-6, which diminishes LTP and limits memory acquisition [63], was not affected by any of the treatments in either genotype (Figure S2a). Furthermore, TNF-α levels were not changed by either CSDS or diet (Figure S2b). These results suggest that the detrimental impact of chronic stress on hippocampal function/synaptic plasticity is not associated with increased neuroinflammation, which is known to affect synaptic plasticity [64].

### 3.7. Effect of Stress and Diet on Hippocampal Fatty Acid and Oxylipins Composition

**Figure 3:**
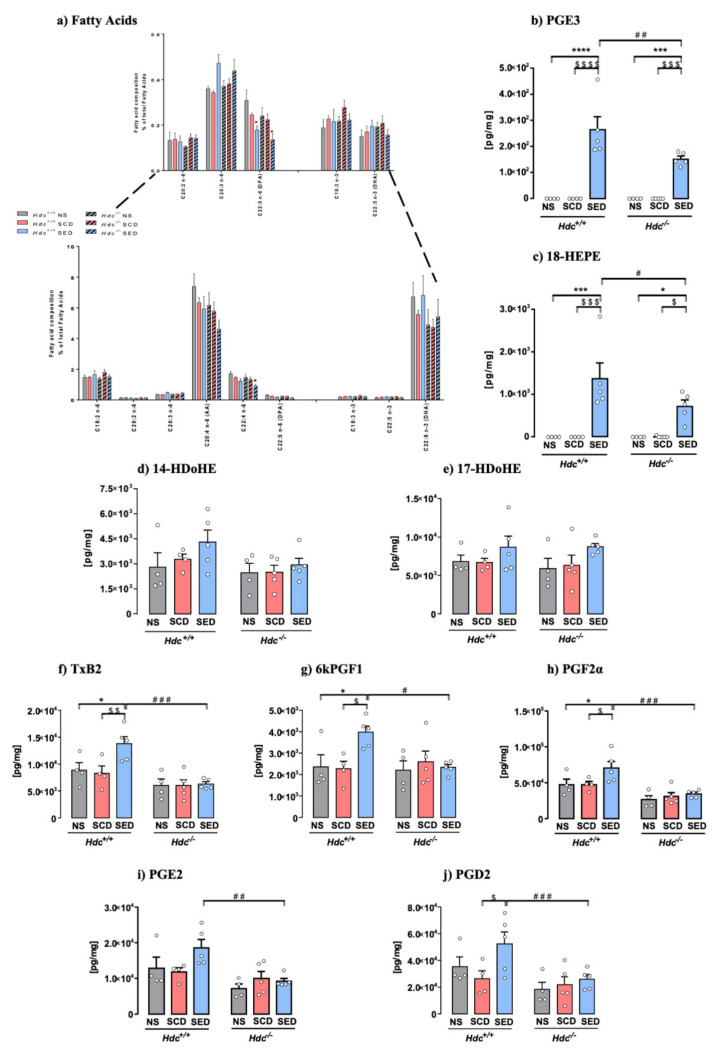
Fatty acid and oxylipins composition of hippocampal tissue sorted from *Hdc*^+/+^ and *Hdc*^−/−^ mice. (**a**) Effect of stress and the enriched diet on total expression of fatty acids. Inset: higher magnification of low expressed fatty acids. (**b**–**j**) Effect of stress and the enriched diet on EPA-, DHA- and AA derived oxylipins in the hippocampus of *Hdc*^+/+^ and *Hdc*^−/−^ mice. Hippocampal content of EPA metabolites PGE3 (**b**) and 18-HEPE (**c**). Tissue content of DHA metabolite 14-HDoHE (**d**) and 17-HDoHE (**e**). (**f**–**j**) Effect of stress and the enriched diet on AA metabolites. Data are expressed as means ± S.E.M. of 4–5 samples per experimental group **** *p* < 0.0001; *** *p* < 0.001, * *p* < 0.05 vs respective NS mice; ^$$$$^ *p* < 0.0001, ^$$$^ *p* < 0.001, ^$$^ *p* < 0.01, ^$^ *p* < 0.05, vs respective SCD mice; ^###^ *p* < 0.001, ^##^ *p* < 0.01, ^#^ *p* < 0.05 vs. same treatment of different genotype. NS = non-stressed; SCD = stressed, control diet; SED = stressed, enriched diet.

Fatty acid composition changes in the lipidic profile may affect several cellular and organ functions [14]. Total saturated (SFA), monounsaturated (MUFAs) fatty acids and PUFAs levels were not significantly different among experimental groups, neither was the n-6/n-3 PUFAs ratio (Table S2). The overall lipidic profile of hippocampal tissue was not significantly affected in any of the experimental groups, which is an indication of membrane integrity in all studied conditions. Stress and the enriched diet, though, altered n-6 PUFAs, adrenic (C22:4) and docosapentaenoic acid (DPA, C22:5, the n-6-derived structural equivalent of DHA), which were equally affected in both genotypes, whereas arachidonic acid (AA, C20:4) was not significantly changed (Figure 3a). Analysis of n-3 PUFAs profile showed no significant differences in alpha-linolenic acid (ALA, 18:3), DPA (22:5 n3) and DHA (22:6) in both genotypes, regardless of treatment and diet (Figure 3a). EPA (20:5 n3) was below detection level. In the brain n-6 and n-3 PUFAs undergo enzymatic conversion to generate bioactive lipid mediators (oxylipins). EPA metabolites PGE3 and 18-HEPE, which are pathway markers for the formation of anti-inflammatory resolvin E, were significantly increased in SED mice of both genotypes, although to a lesser extent in *Hdc*^−/−^ mice (Figure 3b,c). DHA-derived oxylipins 17-HDoHE and 14-HDoHE, which are precursors of resolvin D [13] were unchanged (Figure 3d,e). Overall, stress did not produce an inflammatory profile in either genotype, as AA-derived oxylipins were unchanged in SCD *Hdc*^+/+^ and *Hdc*^−/−^ mice, which were unexpectedly increased in stressed *Hdc*^+/+^ mice only (Figure 3f–j). Other oxylipins derived from AA are shown in Table S3 and were not consistently changed by stress or diet in either genotype.

LC-PUFAs are metabolised into oxylipins through the cyclooxygenase (COX), lipoxygenase (5-LOX and 12/15-LOX) and cytochrome P450 (CYP) pathways. The effect of stress and diet on these enzymes in the hippocampus of the two genotypes are shown in Figure S2. Stress did not augment the expression of these enzymes in either genotype, however, SED *Hdc*^+/+^ mice trended towards increased 5-LOX (Figure S2c) and showed a significant increase of 12-LOX mRNA levels with respect to NS mice (Figure S2d). The other enzymatic pathways were unchanged (Figure S3e–g).

### 3.8. Stress and Diet Modify Microbiota Composition Differently in Hdc^+/+^ and Hdc^−/−^ Mice

**Figure 4:**
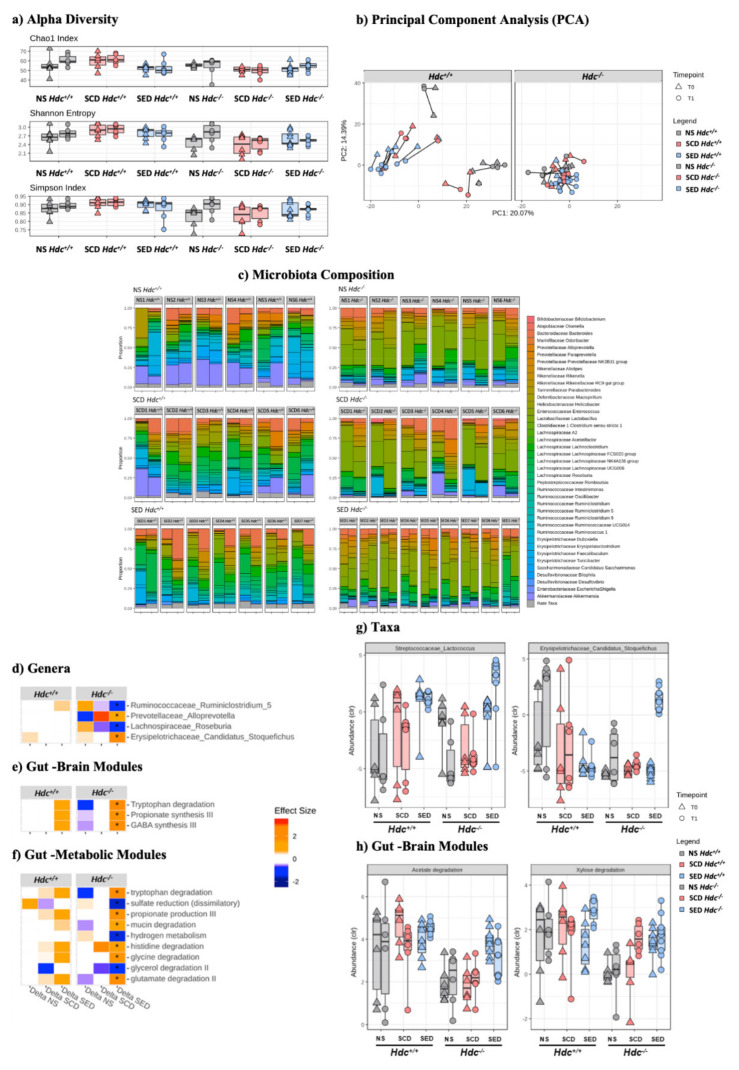
Effect of stress and enriched diet on microbiome. (**a**) Alpha diversity. Non-parametric data are represented as median with inter-quartile ranges (IQR) and min/max values as error bars. Mann–Whitney-U post hoc Bonferroni–Holm. (**b**) PCA showing the microbiome compositions of *Hdc*^+/+^ (left panel) and *Hdc*^−/−^ (right panel) mice, before and after the 10-day chronic stress period. Lines link the same animal over time, showing the trajectory and distance travelled in time. (**c**) Bar plot representation of microbiota composition and response to treatment of *Hdc*^+/+^ and *Hdc*^−/−^ mice of the different experimental groups. (**d**–**h**) Chronic social stress and the enriched diet shape the gut microbiome profile of *Hdc*^+/+^ and *Hdc*^−/−^ mice. Heatmap for Genera (**d**) Gut Brain Modules and (**e**) Gut Metabolic Modules (**f**) differentially altered by treatment. Colours depict effect size, with blue (negative) indicating higher abundances pre-treatment and red (positive) indicating higher abundances post-treatment. * q < 0.1. Relative abundance of (**g**) taxa and (**h**) Gut Brain Modules that displayed an interaction effect in *Hdc*^−/−^ SCD mice and treatment. Benjamini–Hochberg adjusted q < 0.1 for all cases. Data were collected from 5 mice per experimental group.

We did not record an effect of stress or diet in either genotype as measured by alpha diversity in either the Chao1, Shannon or Simpson indices (Figure 4a). However, *Hdc*^−/−^ mice showed a markedly decreased species richness compared to wild type mice, particularly when comparing SCD groups (Figure 4a). To evaluate differences in taxonomic profiles between experimental groups, beta diversity was analysed by using principal component analysis (PCA). As shown in Figure 4b, a clear cluster separation of *Hdc*^+/+^ mice samples with respect to *Hdc*^−/−^ mice was found, regardless of treatment.

The two genotypes exhibited a significant difference in microbiota composition (PERMANOVA; *p* < 0.001, R = 0.102) and response to treatment (PERMANOVA; *p* = 0.027, R = 0.029) suggesting that the absence of host-derived histamine affected the microbiota profile (Figure 4c and Figure S3a). Significant differences between genotypes were also found in predicted gut–brain (Figure S3b) and gut–metabolism modules (Figure S3c). Stress had a minor effect on genus composition of either genotype (Figure S3d). The n-3 LC-PUFA+VitA enriched diet changed the gut microbiota composition of *Hdc*^−/−^ SED mice when compared with *Hdc*^−/−^ SCD mice (Figure 4d and Figure S3d) in line with our previous observations [12]. Strikingly, in terms of differentially abundant genera, the effect was much less prominent in *Hdc*^−/−^ then *Hdc*^+/+^ mice (Figure 4c and Figure S3). Furthermore, there were unique functional profiles associated with the two genotypes (Figure S3b,c,e,f). Among the metabolic functions which pertain to the gut–brain axis, we found enriched categories related to tryptophan degradation and GABA synthesis in SED *Hdc*^−/−^ mice (Figure 4e). Interestingly, tryptophan and histidine degradation were significantly enriched in SED *Hdc*^−/−^ mice (Figure 4f). We also recorded a discrepancy of the enriched diet depending on genotype. For instance, two genera did not exhibit any change in SED *Hdc*^+/+^ compared to a significantly increased abundance in SED *Hdc*^−/−^ (Figure 4g). There was also a similar effect recorded in two functional modules (Figure 4h). This suggests that the reaction of the gut microbiome to chronic stress is dependent on the capacity of the host to produce histamine.

## 4. Discussion

The benefits of a balanced diet extend well beyond mere physical fitness, as adequate nutrition is fundamental for cognitive and emotional status and metabolic equilibria. In our study we used a diet enriched with n-3 LC-PUFAs and vitamin A, a combination of nutrients which we demonstrated being sufficient to prevent the deleterious cognitive decline induced by social instability stress during rats juvenile period [12]. The novel finding of our study is the obligatory role of the histaminergic system to allow the enriched diet to provide a protective effect against the insults of stress. The absence of host histamine synthesis played a key role, not only abrogating the pro-cognitive actions of the diet, but it also prevented the effects of diet on hippocampal neuronal plasticity, hippocampal oxylipin and cytokine profile, and/alongside diet-induced microbiota modifications.

One interesting aspect of our results is that chronic stress per se deteriorated the cognitive performance of wild type and histamine-deficient mice similarly; both genotypes exhibited short-term memory impairment and social avoidance because of the 10 days of social defeat stress. This suggests that the inhibition of histamine synthesis does not affect the maladaptive behavioural response to chronic stress. However, absence of histamine renders stressed mice irresponsive to the effects of the enriched diet.

The short-term memory deficits of both SCD *Hdc*^+/+^ and *Hdc*^−/−^ mice correlated well with malfunctions of the hippocampal CA1 region. CSDS induced cognitive impairment along with increased magnitude of LTP as measured ex vivo in the hippocampal slices. Histamine-deficient mice showed normal basal synaptic transmission and synaptic plasticity in the Schaffer-CA1 pathway, as previously reported by others using similar patterns of stimulation [65]. In our paradigm, mice were sacrificed after completing the social avoidance test, hence the ex vivo recording in the CA1 region expresses the residual hippocampal synaptic plasticity available at that moment. Acute stress, such as a foot shock, produces an occlusion of LTP measured ex vivo in CA1 [66], probably because of information overload and acute activation of plasticity mechanisms in vivo [67]. We hypothesise that the chronic stress-induced cognitive deficits are caused by dysfunctional triggering of endogenous synaptic plasticity mechanisms, such as long-term depression [68], which would be recorded as an increase in LTP magnitude ex vivo. Indeed, it was reported that in rats chronic stress compromised inhibitory post-synaptic currents of hippocampal pyramidal neurons and diet supplementation with n-3 LC-PUFA restored GABAergic function [69]. The enriched diet then, may allow experience induced LTP to overcome the alterations imposed by the chronic stress. Our results provide evidence of some of the cellular mechanisms that are at play to restore cognitive and homeostatic processes which are disturbed by stress.

It is known that n-3 PUFAs are involved in the organisation of plasma membranes [70] that might affect the physiology of hippocampal neurons. In our study, the enriched diet increased the expression of synaptophysin, which is implicated in synaptic neurotransmission, and of IL-1β levels in the hippocampus of *Hdc*^+/+^ mice, and this may be one of the key factors to restore LTP magnitude in vivo. Indeed, IL-1β was shown to be necessary for LTP induction in vivo [64]. We take these results as an indication of synaptic plasticity recovery from stress. Notwithstanding the difficulty in establishing a causal correlation between the changes in neurochemical biomarkers and recovery of learning capability of mice, it is attractive to conceive that the enriched diet promotes changes in IL-1β and synaptophysin, presumably with other plasma membrane components such as receptors and channels, that concur in re-establishing physiological mechanisms of synaptic plasticity in stressed *Hdc*^+/+^ mice. It is of interest that in vivo the presence of histamine is permissive for such recovery. Indeed, the enriched diet normalised synaptic plasticity only in mice with a functioning histaminergic system. The puzzling questions remain on why (and how) dietary components or microbiota metabolites are inefficient in *Hdc*^−/−^ mice. The n-3 LC-PUFAs are mainly supplied by the diet and the systemic circulation delivers them to the brain where they accumulate and/or are metabolised into oxylipins. EPA (one of the enriched diet components) for instance, does not accumulate in the brain, but is oxidised into oxylipins such as 18-HEPE [13]. 18-HEPE has been reported to be increased in the blood of depressed patients after dietary supplementation with EPA+DHA; and 18-HEPE-derived resolvins display antidepressant effect in rodents [71]. Most likely, the failure of the diet to protect histamine deficient mice from the insults of chronic stress is not attributable to poor absorption or altered metabolism of nutrients in the hippocampus, as EPA-derived oxylipins 18-HEPE and PGE3 were increased in the hippocampus of both SED *Hdc*^+/+^ and, although to a significantly less extent, *Hdc*^−/−^ mice. Nonetheless, the different expression of oxylipins may differentially contribute to the beneficial effects of n-3 LC-PUFAs as these molecules possess anti-inflammatory and pro-resolving properties [72] and are also potent regulators of synaptic plasticity [73]. Based on previous observations, we suggested that histamine-deficient mice are incapable of elaborating on appropriate behavioural responses which are unlocked from their stereotypical repertoire [7]. In this regard, the enriched diet appears to be insufficient for *Hdc*^−/−^ mice to maintain normal behavioural responses in the face of challenging stressful stimuli.

We cannot exclude that peripheral mechanisms as well, are at play and contribute to the malfunctioning of a histamine deficient brain. The viscera communicate with the CNS via multiple pathways, such as the vagus nerve and the spinal cord [74], hepatic portal signalling [75] and the blood stream [76]. Signals form visceral organs and nutrients access the CNS to provide information on general physiological conditions in order to maintain homeostasis, and the microbiota is a key factor in the communication axis between the gut and brain [34]. As our previous results suggested that the intestinal microbiome may be involved in the stress ameliorating effect(s) of the diet [12] we used the same approach in this present work. We found a striking difference between *Hdc*^+/+^ and *Hdc*^−/−^ mice that extends to the composition of the gut microbiota under control conditions and in response to stress and diet. The absence of host histamine profoundly affected the faecal microbiota composition and its response to stress with respect to wild type mice. Considering the nature of the *Hdc*^−/−^ mice, this is not surprising. Indeed, histamine is known to play a major regulatory role in the microbiome [77]. Host genetics are also known to shape the microbial composition and function [78]. It is clear that a change in host genetics can undoubtedly affect the microbial environment of the gut. Our study did not identify a clear effect of stress on the microbiome of either genotype in terms of differential abundance. From the available literature we expected this to be a consistent effect of CSDS on the microbiome [12]. Nevertheless, it is likely that CSDS influenced the microbiome given the clear effects on beta diversity. As expected from our previous studies [12], the enriched diet had a significant effect on the microbiome on both diversity and relative abundance. This was particularly evident for the gut-metabolic modules, which represent the capacity of the microbiome to metabolise different types of substrates. For instance, we recorded substantial changes in metabolic signatures in the NS vs SED mice of either genotype at baseline (T0). Furthermore, >86% of the significantly altered functional modules were upregulated in the enriched diet (SED) samples compared to the non-stressed control diet (NS) group. This implies that the enriched diet increases the functional capacity of the microbiome overall. This is reflected by the increase in metabolic potential for amino acid and sugar metabolism. In general, functions that are upregulated in a microbiome are considered to likely be more important for the homeostasis of that microbiome in a given environment [79]. In this study we used the Piphillin framework, which reflects shotgun sequencing datasets reasonably well for mammalian samples [80], to infer microbial function. Indeed, several 16S rRNA gene studies have extended our ability to infer the functional contribution of individual bacterial community members by focusing on groups of genes associated with the synthesis of compounds which have the potential to interact with the human nervous system [51]. However, the functional inference method used here is limited by the database it employs and given the 16S nature of our data, we have no conclusive evidence of the functional capacities presented here that would require further confirmatory investigation. In summary, the enriched diet did not rescue the negative behavioural effects of stress in *Hdc*^−/−^ mice, in contrast to *Hdc*^+/+^ animals. Due to the substantial effect of histamine absence on the microbiome, even at baseline, we cannot ascertain to what extent the microbiome partakes in preventing the stress-induced alterations in wild type mice fed the enriched diet. Histamine is a ubiquitous molecule and given that lack of histamine causes systemic alterations to the overall physiology of the animal, it is difficult to resolve this issue. However, our results suggest a potential cross talk between the peripheral histaminergic system and the gut microbiota which is compromised in *Hdc*^−/−^ mice as these animals have minimal histamine release from mast cells and basophils [81].

Taken together, our results provide strong evidence for the beneficial role of a diet supplemented with n-3 LC-PUFAs and vitamin A, confirming our findings obtained in a rat model of juvenile stress [12]. We suggest that the histaminergic system has an indispensable role within the gut–brain axis, as it allows the unfolding of the beneficial effects of diet supplementations. The obligatory role of brain histamine adds further relevance to our hypothesis that histaminergic neurons provide a hub that integrates peripheral and central inputs to orchestrate the appropriate behavioural output.

Many questions remain unanswered that constitute the limitation of our work and deserve further studies. For instance, nothing is known of the interplay between microbiota and host genetics, which histaminergic receptors are involved, how the absence of histamine affects the expression of oxylipins and synaptic components such as synaptophysin. Furthermore, the degree of difference was much larger than anticipated, which limits our capacity to investigate directionality of this microbiome–genome interaction specifically. Nonetheless, we believe that our findings open new scenarios in the field of nutritional neuropsychopharmacology and in understanding the impact of histamine and histaminergic compounds on stress-induced maladaptive responses. As markers of histaminergic dysregulation are found in several neuropsychiatric disorders [82], presumably an inefficient histaminergic system that cannot elaborate peripheral or internal signals may contribute to the onset or progression of neural diseases, or to the lack of efficacy of therapeutic interventions. Histamine H_1_ receptor antagonists are among the most used drugs worldwide and our research may provide knowledge on how to improve their pharmacological profile and to reveal unexplored therapeutic applications.

## Data Availability

Study protocol and all data collected for the study, including raw data and data analysis will be made available to others upon request. All data will be available upon publication of the manuscript, by contacting the corresponding author.

## Supplementary Materials

The following supporting information can be downloaded at: https://www.mdpi.com/article/10.3390/ijms23020862/s1.
