# Supplementary material for: Diet Prevents Social Stress-Induced Maladaptive Neurobehavioural and Gut Microbiota Changes in a Histamine-Dependent Manner
Source: Int J Mol Sci. 2022 Jan 13;23(2):862. doi: 10.3390/ijms23020862 (PMC8775792; doi:10.3390/ijms23020862)
Supplement: Supplementary file 1 [file ijms-23-00862-s001.zip › Supplementary Materials Costa Rani IJMS.pdf]

Supplementary Materials

Methods

Chronic Social Defeat Stress the procedure consisted of the introduction of an experimental mouse of either genotype in the cage of a CD-1 aggressor until the first aggression and before wounding occurred. Mice were then separated by a transparent, perforated Plexiglas barrier to enable constant sensory exposure. After 2 hrs, the plexiglass separator was removed, and the second attack occurred. The procedure was repeated daily for 10 consecutive days from PND56 to PND65, changing CD1 aggressor every day and at different timing during the day to avoid habituation. The stress protocol included overcrowding sessions that consisted of 6/8 experimental mice placed together in a standard holding cage (33 × 15 × 13 cm) for 24 h on days 3-4, 8-9 with diet and water available *ad libitum.*

Western blot analysis. The pooled structures (left and right) were homogenized in 200 μL ice-cold lysis buffer containing protease and phosphatase inhibitors (50mM TrisHCl (pH 7.5), 50mM NaCl, 10mM EGTA, 5mM EDTA, 2mM NaPP, 4mM PNFF, 1mM Na3VO4, 1.1mM PMSF, 20 μg/μL Leupeptin, 50 μg/μL Aprotinin, 0.1% SDS) and centrifuged at 12000 rpm at 4 °C for 15 minutes. The supernatant was collected, and total protein levels were quantified using the Pierce BCA Protein Assay (Thermo Scientific, USA). Homogenates were diluted in a mix of lysis buffer and loading buffer 2x (50mM Tris pH = 6.8, 100mM DTT, 10% Glycerol, 1% Bromophenol Blue, and 2% SDS) and boiled for 10 minutes. Aliquots containing 15 μg of total proteins, for detection of synaptophysin, were resolved by electrophoresis on a 10% SDS-polyacrylamide gel (SDS-PAGE) and transferred onto polyvinylidene difluoride (PVDF) membranes (Immobilon Transfer Membranes, Millipore, USA). Blots were blocked in Tris-buffered saline, pH 7.6 containing 0.1% of Tween 20 (TBS-T) and 5% non-fat dry milk (Bio-Rad Laboratories, USA) for 2 h at room temperature and then incubated overnight at 4°C with antibodies against synaptophysin (1:5000 Thermo Fisher Scientific Cat# PA1-1043, RRID:AB_2199026) or tubulin-alpha monoclonal antibody HRP-conjugated (1:10000 Proteintech Cat# HRP-66031, RRID:AB_2687491) all diluted TBS-T containing 5% non-fat dry milk. Immunodetection for antibodies against synaptophysin was performed with secondary antibodies (anti-rabbit IgG conjugated to horseradish peroxidase, Cell Signaling Technology Cat# 7074, RRID:AB_2099233) diluted 1:5000 in TBS-T containing 1% of non-fat dry milk. Membranes were washed in TBS-T and then reactive bands were detected using enhanced chemiluminescence (Luminata Crescendo, Millipore, USA). Quantitative densitometric analysis was performed using the QuantityOne analysis software (Bio-Rad). For each sample, a ratio of Synaptophysin/Tubulin densities was calculated and then all the

individual rates were expressed as a percentage of the average of ratios obtained from control groups.

Real-Time PCR. cDNAs for (IL-1β, IL-6, TNF-α, 5-LOX, 12-LOX, COX2, CYP1A1, soluble hydrolase (EPHX2) and a housekeeping gene (GAPDH) were amplified by PCR using an oligonucleotide probe with a 5′ fluorescent reporter dye (6-FAM) and a 3′ quencher dye (NFQ). PCR program consisted of 40 cycles of 95 °C for 15 s and 60 °C for 1 min. Fluorescence will be measured using an AB 7500 Real-Time PCR system (Applied Biosystems, Foster city, CA). The data of real time PCR are expressed as Relative Quantification.

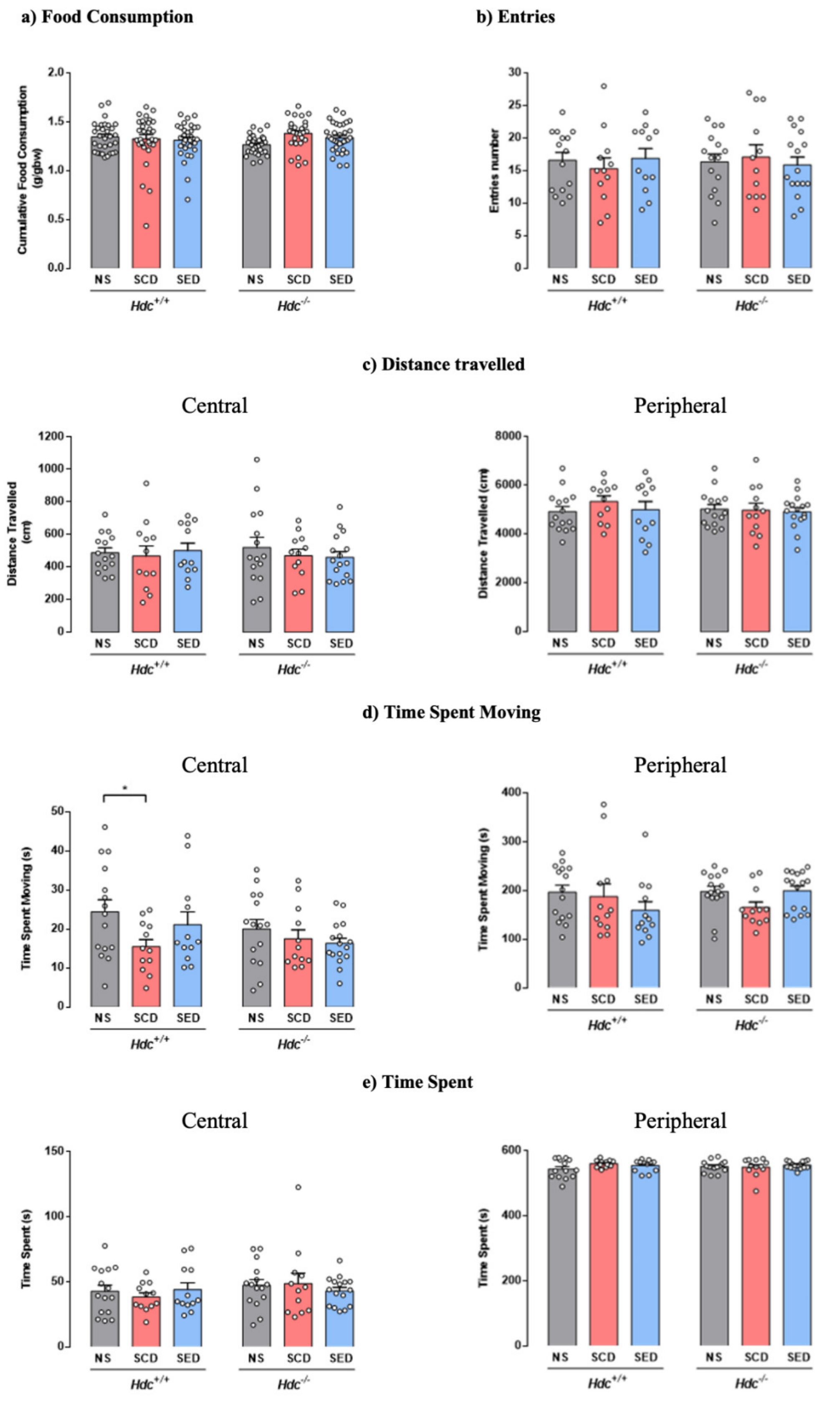

Figure S1. Effect of stress and enriched diet on food consumption, locomotor activity and anxiety-like behaviour. (a) Cumulative food consumption. (b) Number of entries in the central area of the

arena. (c) Distance travelled in central or peripheral areas of the arena. (d) Time mice spent moving in the central or peripheral areas of the arena. (e) Time mice spent within the central or peripheral areas of the arena. Data are expressed as means ± s.e.m. of 27-34 samples per experimental group in panel (a), and 12-16 samples for panles (b-e). *P < 0.05. NS = non-stressed; SCD = stressed, control diet; SED = stressed, enriched diet.

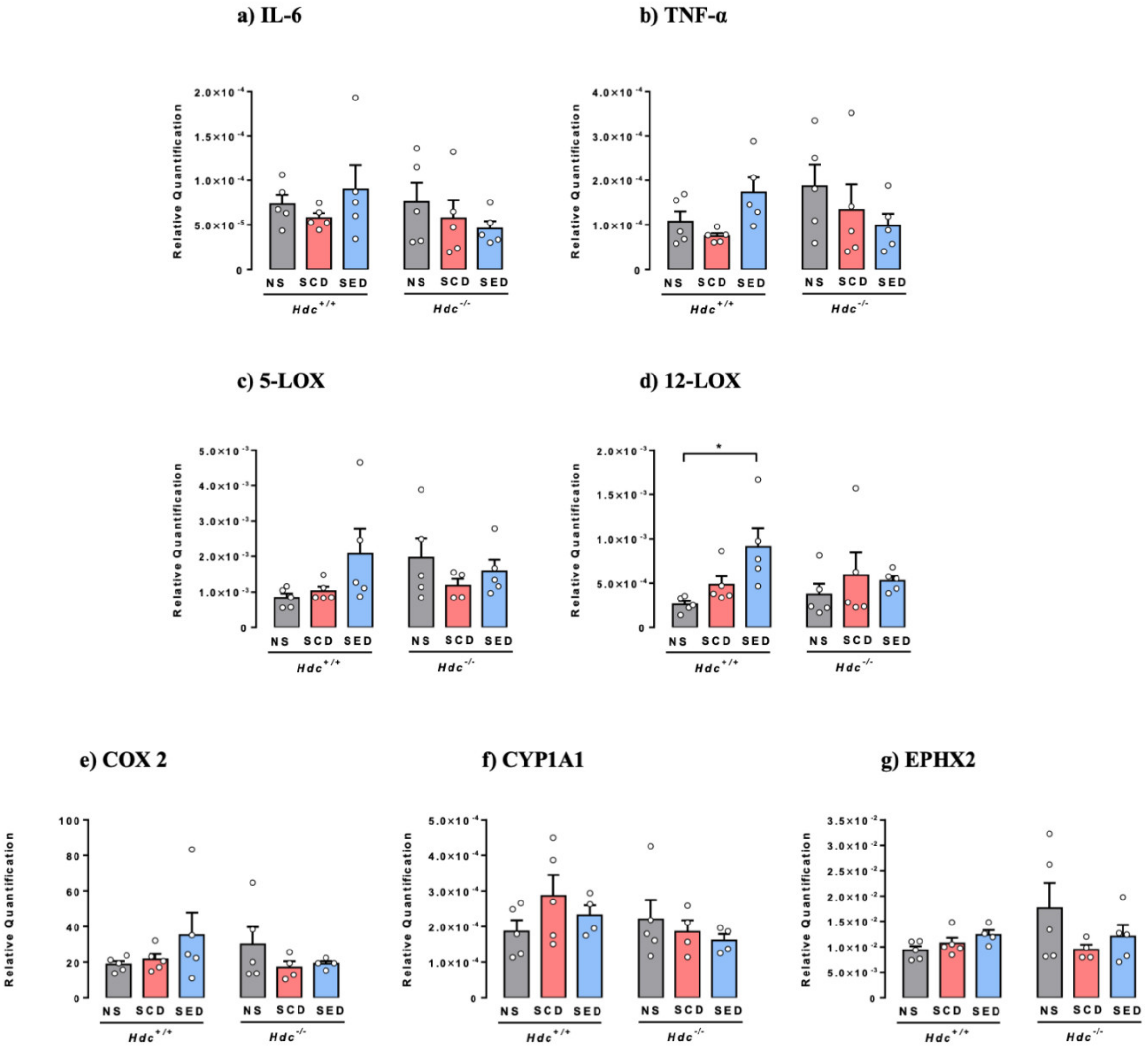

Figure S2. Effect of stress and the enriched diet on the expression of cytokines and enzymes involved in fatty acid metabolism in the hippocampus of *Hdc*$^{+/+}$ and *Hdc*$^{-/-}$ mice. (a-g) Densiometric quantification in hippocampal tissue. IL-6 = interleukin-6; TNF-α = tumor necrosis factor α; LOX = lipoxygenase; COX-2 = Cyclooxygenase 2; CYP1A1 = Cytochrome 1A1; EPH = epoxide hydrolases. Results are expressed as means ± s.e.m. of 5 samples per

experimental group in panel (a,b) and 4-5 samples in panels (c-g). *P < 0.05 vs respective NS mice. NS = non-stressed; SCD = stressed, control diet; SED = stressed, enriched diet.

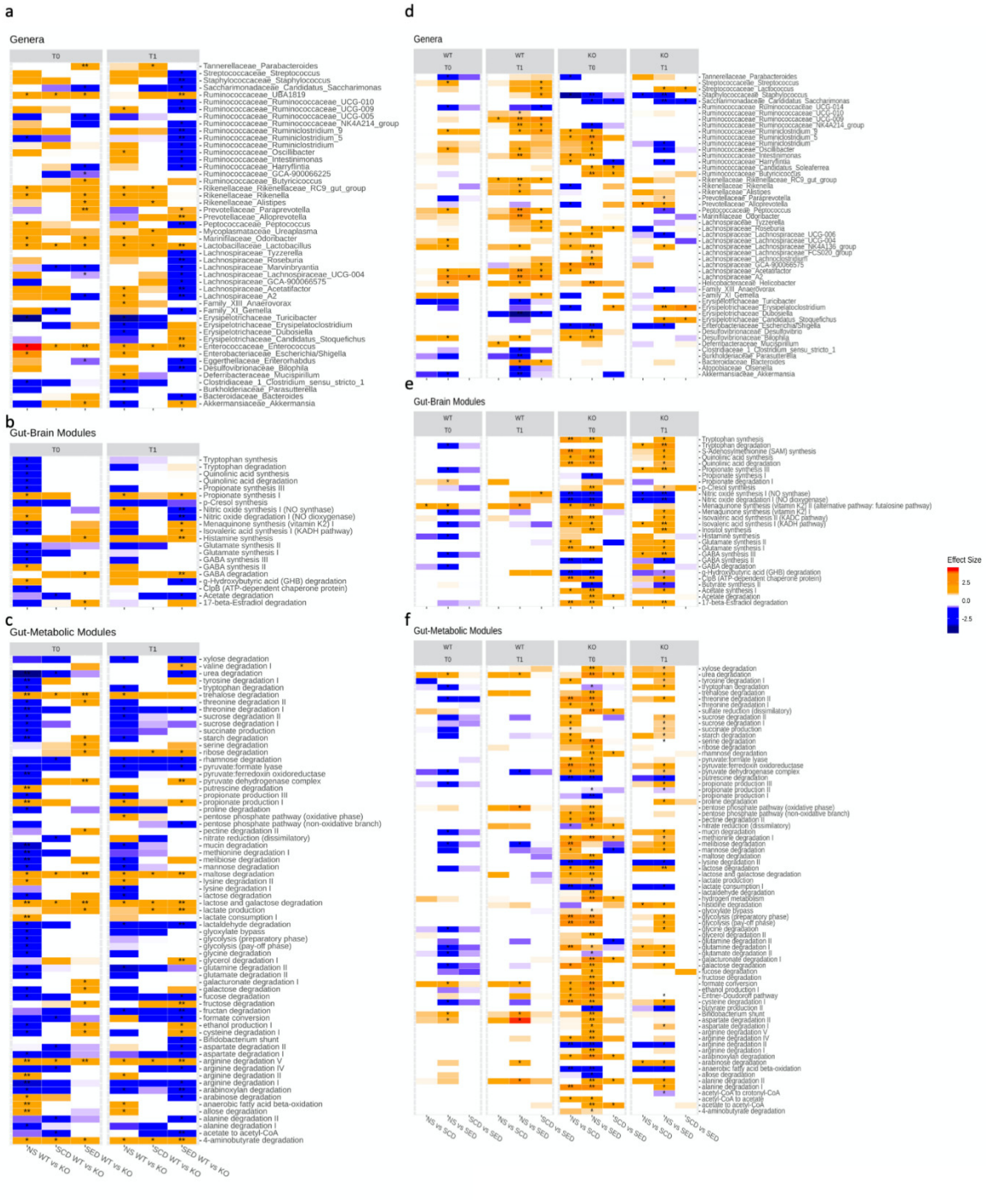

Figure S3. Genotype and diet shape the gut microbiome. (a, d) Changes in gut microbiome composition (genera) before (T0) and after (T1) chronic stress. (b, e) Changes in predicted gut-brain metabolic pathways and (c, f) gut-metabolic pathways. Changes in each treatment group are depicted at the bottom of each column. WT = *Hdc*$^{+/+}$ mice; KO = *Hdc*$^{-/-}$ mice; *P < 0.1, **P < 0.01, post hoc Benjamini–Hochberg test. Data were collected from 5 samples.

Table S1. Composition of the control diet and n3 LC-PUFA+VitA enriched diet (from Ssniff Spezialdi.ten GmbH).

		Control Diet	EPA/DHA
		**5,000 IU/kg Vit. A**	**45,000 IU/kg Vit. A**
Product No.		*SPP12-E020*	*S9912-E022*
Casein	%	200.000	200.000
Corn Starch, pre-gelatinized	%	374.984	374.904
Maltodextrin	%	150.000	150.000
Sucrose	%	100.000	100.000
Cellulose	%	50.000	50.000
L-Cystine	%	0.3000	0.3000
Mineral & trace element premix	%	60.000	60.000
Vitamin premix, AIN *	%	10.000	10.000
TBHQ (t-butylhydroquinone)	%	0.0014	0.0014
Vitamin A	%	0.0002	0.0082
Choline Cl	%	0.2000	0.2000
HO Sunflower Oil	%	19.000	14.000
Palm Oil	%	22.000	-
Canola Oil	%	0.8000	-
EPA oil	%	-	25.000
Safflower oil	%	-	0.4000
Soybean Oil	%	0.1000	0.7000
Crude protein	%	17.6	17.6
Crude fat	%	5.1	5.1
Crude fibre	%	5.0	5.0
Crude ash	%	5.4	5.4
Starch	%	36.1	36.1
Sugar	%	11.1	11.1
Fatty Acids			
C 12:0	%	-	0.01
C 14:0	%	0.03	0.20
C 16:0	%	1.12	0.65
C 18:0	%	0.18	0.18
C 20:0	%	0.02	0.02
C 16:1	%	0.01	0.21
C 18:1	%	2.94	1.72
C 18:2	%	0.55	0.79
C 18:3	%	0.09	0.06
C 20:5 *n3*	%	-	0.44
C 22:5 *n3*	%	-	0.05
C 22:6 *n3*	%	-	0.30
Physiological fuel value	MJ/kg	15.5	15.5
Protein	kcal%	19	19
Fat	kcal%	13	13
Carbohydrates	kcal%	68	68

Table S2. Effect of chronic stress and the enriched diet on total fatty acid composition in the hippocampus of *Hdc*$^{+/+}$ and *Hdc*$^{-/-}$mice. Total levels of fatty acids were not significantly different among genotypes regardless of stress or diet. The n6/n3 PUFA ratio was not affected as well. Data are expressed as percentage of total fatty acids in 4-5 mice per experimental group. NS non-stressed, control diet; SCD stressed control diet; SED stressed enriched diet.

	% of total fatty acids						Statistical effects								
	Hdc $^{+/+}$			*Hdc* $^{-/-}$			Conditiong			Genotype			Interaction		
	NS	SCD	SED	NS	SCD	SED	F (DFn, DFd)	P value	P value summary	F (DFn, DFd)	P value	P value summary	F (DFn, DFd)	P value	P value summary
**12:0**	0,245 ± 0,1334	0,9275 ± 0,172	0,548 ± 0,0887	0,0975 ± 0,0284	0,104 ± 0,0204	0,148 ± 0,0373	F (2, 21) = 6,776	P = 0,0054	**	F (1, 21) = 37,75	P < 0,0001	****	F (2, 21) = 6,748	P = 0,0055	**
**14:0**	0,235 ± 0,0357	0,43 ± 0,128	0,366 ±0,0889	0,142 ± 0,0103	0,162 ± 0,0244	0,23 ± 0,0164	F (2, 21) = 1,776	P = 0,1937		F (1, 21) = 9,838	P = 0,0050	**	F (2, 21) = 0,9744	P = 0,3938	
**15:0**	0,1575 ± 0,0357	0,320 ± 0,0703	0,21 ± 0,0752	0,11 ± 0,0141	0,116 ± 0,0136	0,138 ± 0,0233	F (2, 21) = 1,539	P = 0,2379		F (1, 21) = 7,954	P = 0,0103	*	F (2, 21) = 1,592	P = 0,2270	
**16:0**	25,19 ± 0,740	26,21 ± 0,841	26,77 ± 1,085	24,08 ± 1,027	24,34 ± 1,424	26,76 ± 0,866	F (2, 21) = 2,160	P = 0,1403		F (1, 21) = 1,304	P = 0,2664		F (2, 21) = 0,4089	P = 0,6696	
**17:00**	0,25 ± 0,0334	0,302 ± 0,0175	0,276 ± 0,0425	0,25 ± 0,0227	0,28 ± 0,0251	0,292 ± 0,022	F (2, 21) = 1,043	P = 0,3699		F (1, 21) = 0,008047	P = 0,9294		F (2, 21) = 0,2250	P = 0,8004	
**18:0**	23,72 ± 0,415	22,71 ± 0,581	23,03 ± 0,576	25,98 ± 0,599	26,36 ± 0,398	25,87 ± 0,871	F (2, 21) = 0,2282	P = 0,7979		F (1, 21) = 33,62	P < 0,0001	****	F (2, 21) = 0,6070	P = 0,5543	
**20:0**	0,3 ± 0,0374	0,362 ± 0,0628	0,458 ± 0,0678	0,282 ± 0,0510	0,276 ± 0,0443	0,234 ± 0,0322	F (2, 21) = 0,5672	P = 0,5756		F (1, 21) = 6,768	P = 0,0167	*	F (2, 21) = 2,144	P = 0,1421	
**22:0**	0,255 ± 0,0504	0,327 ± 0,0239	0,318 0,0416	0,342 ± 0,00946	0,346 ± 0,0201	0,336 ± 0,0378	F (2, 21) = 0,6225	P = 0,5462		F (1, 21) = 2,179	P = 0,1547		F (2, 21) = 0,6448	P = 0,5349	
**24:0**	0,36 ± 0,0652	0,435 ± 0,0429	0,46 ± 0,0200	0,575 ± 0,0166	0,554 ± 0,0391	0,588 ± 0,0465	F (2, 21) = 0,9493	P = 0,4030		F (1, 21) = 20,99	P = 0,0002	***	F (2, 21) = 0,7825	P = 0,4701	
Total saturated fatty acids	50,715 ± 0,901	52,03 ± 1,396	52,44 ± 1,868	51,86 ± 1,650	52,53 ± 1,654	54,6 ± 1,621	F (2, 21) = 0,9703	P = 0,3953		F (1, 21) = 0,9334	P = 0,3450		F (2, 21) = 0,1431	P = 0,8675	
**16:1n-9**	0,2075 ± 0,0232	0,28 ± 0,0802	0,244 ± 0,0143	0,1875 ± 0,0118	0,204 ± 0,0329	0,222 ± 0,0274	F (2, 20) = 0,9805	P = 0,3924		F (1, 20) = 2,152	P = 0,1579		F (2, 20) = 0,4422	P = 0,6487	
**16:1n-7**	0,445 ± 0,0185	0,562 ± 0,106	0,532 ± 0,0392	0,395 ± 0,0218	0,540 ± 0,168	0,672 ± 0,115	F (2, 21) = 1,599	P = 0,2257		F (1, 21) = 0,07073	P = 0,7929		F (2, 21) = 0,5153	P = 0,6047	
**18:1t**	0,1375 ± 0,0103	0,217 ± 0,0644	0,16 ± 0,0219	0,217 ± 0,0184	0,236 ± 0,0169	0,198 ±0,0285	F (2, 21) = 1,688	P = 0,2090		F (1, 21) = 3,338	P = 0,0819		F (2, 21) = 0,4991	P = 0,6141	
**18:1n-9**	17,1325 ± 0,774	17,315 ± 0,165	17,808 ± 0,600	19,1 ± 0,544	18,74 ± 0,431	16,056 ± 0,463	F (2, 21) = 0,3426	P = 0,7138		F (1, 21) = 12,73	P = 0,0018	**	F (2, 21) = 0,2417	P = 0,7874	
**18:1n-7**	3,6 ± 0,103	3,315 ± 0,0899	3,288 ± 0,0996	4,32 ± 0,159	4,166 ± 0,0528	3,768 ± 0,109	F (2, 21) = 8,527	P = 0,0019	**	F (1, 21) = 63,88	P < 0,0001	****	F (2, 21) = 1,721	P = 0,2031	
**20:1n-9**	1,11 ± 0,146	1,01 ± 0,0272	1,042 ± 0,0680	1,26 ± 0,0454	1,25 ± 0,0981	1,08 ± 0,0579	F (2, 20) = 1,095	P = 0,3538		F (1, 20) = 3,879	P = 0,0629		F (2, 20) = 0,6574	P = 0,5290	
**20:1n-7**	0,265 ± 0,0328	0,24 ± 0,0108	0,256 ± 0,016	0,342 ± 0,00750	0,33 ± 0,0281	0,278 ± 0,0183	F (2, 21) = 1,484	P = 0,2495		F (1, 21) = 13,12	P = 0,0016	**	F (2, 21) = 1,533	P = 0,2391	
**22:1n-9**	0,5075 ± 0,171	1,225 ± 0,560	0,488 ± 0,123	0,515 ± 0,0340	0,778 ± 0,196	0,454 ± 0,0129	F (2, 21) = 3,176	P = 0,0624		F (1, 21) = 0,6715	P = 0,4217		F (2, 21) = 0,5637	P = 0,5775	
**24:1n-9**	0,4325 ± 0,0803	0,443 ± 0,00882	0,42 ± 0,0386	0,68 ± 0,0147	0,638 ± 0,0884	0,558 ± 0,0868	F (2, 20) = 0,5418	P = 0,5900		F (1, 20) = 11,25	P = 0,0032	**	F (2, 20) = 0,3213	P = 0,7289	
Total monounsaturated fatty acids	23,8375 ± 1,224	26,135 ± 2,108	24,238 ± 0,912	27,017 ± 0,813	26,882 ± 0,431	26,29 ± 0,748	F (2, 21) = 0,7872	P = 0,4681		F (1, 21) = 5,011	P = 0,0361	*	F (2, 21) = 0,5937	P = 0,5613	
Total ω-6 fatty acids	11,3875 ± 0,879	10,015 ± 0,455	9,598 ± 0,773	9,755 ± 0,998	9,694 ± 0,604	7,744 ± 0,734	F (2, 21) = 3,270	P = 0,0581		F (1, 21) = 4,197	P = 0,0532		F (2, 21) = 0,6089	P = 0,5533	
Total ω-3fatty acids	7,06 ± 0,917	5,97 ± 0,267	7,22 ± 1,260	5,295 ± 0,966	5,22 ± 0,425	5,806 ± 1,123	F (2, 21) = 0,5031	P = 0,6118		F (1, 21) = 2,864	P = 0,1054		F (2, 21) = 0,1424	P = 0,8681	
Total PUFA	18,585 ± 1,764	16,182 ± 0,677	16,972 ± 2,010	15,24 ± 1,962	15,104 ± 1,059	13,718 ± 1,772	F (2, 21) = 0,4882	P = 0,6205		F (1, 21) = 3,593	P = 0,0719		F (2, 21) = 0,3009	P = 0,7433	
ω-6/ω-3	1,645 ± 0,0861	1,6775 ± 0,0340	1,454 ± 0,1770	1,9325 ± 0,1637	1,882 ± 0,0934	1,456 ± 0,161	F (2, 21) = 4,096	P = 0,0315		F (1, 21) = 2,177	P = 0,1550		F (2, 21) = 0,5965	P = 0,5598	*

Table S3. Effect of chronic stress and diet on arachidonic acid derived oxylipins in the hippocampus of *Hdc*$^{+/+}$ and *Hdc*$^{-/-}$mice. The statistical analysis and significance are also shown in the Table.

Oxylipin	[pg/mg]						Statistical effects								
	Hdc$^{+/+}$			*Hdc*$^{-/-}$			Conditiong			Genotype			Interaction		
	NS	SCD	SED	NS	SCD	SED	F (DFn, DFd)	P value	P value summary	F (DFn, DFd)	P value	P value summary	F (DFn, DFd)	P value	P value summary
15dPGJ2	306,18 ± 46,76	345,25 ± 40,38	470,33 ± 227,40	126,02 ± 27,90	154,03 ± 27,42	164,94 ± 9,34	F (2, 21) = 0,4645	P = 0,6348		F (1, 21) = 6,441	P = 0,0191	*	F (2, 21) = 0,2138	P = 0,8093	
8isoPGA2	974,86 ± 186,91	1044,265 ± 160,20	1595,438 ± 515,47	536,32 ± 86,55	650,23 ± 137,65	603,66 ± 515,47	F (2, 21) = 0,9659	P = 0,3969		F (1, 21) = 8,147	P = 0,0095	**	F (2, 21) = 0,8662	P = 0,4351	
LTB4	73,7725 ± 21,46	57,39 ± 11,49	60,51 ± 19,21	52,58 ± 20,51	45,82 7 8,72	52,62 ± 3,89	F (2, 21) = 0,2777	P = 0,7603		F (1, 21) = 1,203	P = 0,2852		F (2, 21) = 0,1002	P = 0,9051	
LxA4	581,5475 ± 117,58	445,3 ± 60,13	707,66 ± 97,64	602,34 ± 174,54	456,87 ± 122,36	527,54 ± 76,42	F (2, 21) = 1,298	P = 0,2941		F (1, 21) = 0,2874	P = 0,5976		F (2, 21) = 0,5371	P = 0,5923	
14,15-EET	657,3975 ± 123,86	845,6 ± 116,87	1036,79 ± 634,30	620,02 ± 140,0	624,66 ± 88,34	463,72 ± 96,62	F (2, 21) = 0,07298	P = 0,9299		F (1, 21) = 1,198	P = 0,2861		F (2, 21) = 0,3958	P = 0,6781	
11,12-EET	1143,223 ± 359,78	1228,422 ± 195,58	349,8775 ± 55,59	487,99 ± 94,74	531,34 ± 103,48	392,094 ± 136,62	F (2, 20) = 4,921	P = 0,0183	*	F (1, 20) = 8,934	P = 0,0073	**	F (2, 20) = 2,753	P = 0,0879	
8,9-EET	4471,835 ± 734,57	4428,2 ± 328,2	5806,696 ± 1962,25	3452,115 ± 790,64	2820,86 ± 316,94	2500,752 ± 386,79	F (2, 21) = 0,1443	P = 0,8665		F (1, 21) = 5,629	P = 0,0273	*	F (2, 21) = 0,7017	P = 0,5070	
5,6-EET	8548,855 ± 1559,06	8084,272 ± 1112,33	11038,89 ± 1920,23	5549,01 ± 1662,14	4841,12 ± 657,54	5558,138 ± 317,91	F (2, 21) = 1,085	P = 0,3561		F (1, 21) = 13,23	P = 0,0015	**	F (2, 21) = 0,5689	P = 0,5746	
15-HETE	25071,63 ± 3490,41	31849,68 ± 4097,05	35435,61 ± 4615,73	24590,53 ± 5960,72	21134,34 ± 3373,75	21501,79 ± 1248,27	F (2, 21) = 0,4288	P = 0,6569		F (1, 21) = 6,734	P = 0,0169	*	F (2, 21) = 1,529	P = 0,2400	
8-HETE	1981,275 ± 339,59	2073,345 ± 167,14	2259,712 ± 400,81	1474,458 ± 388,69	1290,856 ± 213,08	1433,242 ± 95,53	F (2, 21) = 0,1832	P = 0,8339		F (1, 21) = 8,887	P = 0,0071	**	F (2, 21) = 0,1713	P = 0,8437	
12-HETE	75640,68 ± 42426,33	59929,79 ± 6049,25	88795,48 7 18777,84	22691,42 7 5247,63	46282,88 ± 7826,03	41912,63 ± 13193,59	F (2, 21) = 0,4051	P = 0,6720		F (1, 21) = 5,870	P = 0,0245	*	F (2, 21) = 0,6035	P = 0,5561	
5-HETE	23194,76 ± 3711,08	19647,02 ± 2161,47	25123,47 ± 4186,23	19396,55 ± 5315,62	15578,56 ± 2717,99	17177,67 ± 1042,23	F (2, 21) = 0,7514	P = 0,4840		F (1, 21) = 3,600	P = 0,0716		F (2, 21) = 0,2456	P = 0,7845	
5-oxoETE	75873,34 ± 12942,71	70833,22 ± 8164,00	90893,29 ± 13688,67	36025 ± 9837,78	37777,02 ± 5486,13	52040,46 ± 3725,81	F (2, 21) = 2,073	P = 0,1509		F (1, 21) = 22,60	P = 0,0001	***	F (2, 21) = 0,07206	P = 0,9307	
13-HODE	5586,317 ± 575,01	6184,143 7 780,90	7285,844 ± 1471,50	4697,625 ± 1236,48	6632,394 ± 1779,17	5989,154 ± 662,98	F (2, 21) = 0,8013	P = 0,4620		F (1, 21) = 0,3236	P = 0,5755		F (2, 21) = 0,2767	P = 0,7610	
9-HODE	1610,637 ± 206,77	1674,488 ± 188,37	1694,79 ± 245,37	1862,435 ± 457,19	2381,974 ± 585,21	1875,806 ± 232,76	F (2, 21) = 0,3564	P = 0,7043		F (1, 21) = 1,607	P = 0,2188		F (2, 21) = 0,3088	P = 0,7376	

Table S4. Statistical analysis of data shown in Figures 1–3.

Figure	"n" (per group)	Analysis (post hoc reported in figures)	Factors Analyzed	F ratios		P values
**1B**	27-34	two-way ANOVA (Bonferroni)	Genotype (*Hdc* $^{+/+}$ *vs Hdc* $^{-/-}$)	Interaction	F (2, 179) = 4,804	P=0,0093
			Conditions (NS vs SCD vs SED)	Genotype	F (1, 179) = 3,511	P=0,0626
				Conditions	F (2, 179) = 14,4	P<0,0001
**1C**	27-34	two-way ANOVA (Bonferroni)	Genotype/Conditions	Interaction	F (5, 358) = 31,33	P < 0,0001
			Time (time target absent *vs* time target present)	Gen/Cond	F (5, 358) = 62,39	P < 0,0001
				Time	F (1, 358) = 29,87	P < 0,0001
**1D**	27-34	two-way ANOVA (Bonferroni)	Genotype (*Hdc* $^{+/+}$ *vs Hdc* $^{-/-}$)	Interaction	F (2, 179) = 18,21	P<0,0001
			Conditions (NS *vs* SCD *vs* SED)	Genotype	F (1, 179) = 25,3	P<0,0001
				Conditions	F (2, 179) = 117,9	P<0,0001
**1E**	5-9	two-way ANOVA (Bonferroni)	Genotype/Conditions	Interaction	F (5, 70) = 5,300	P = 0,0003
			Time (time familiar object *vs* time novel object)	Gen/Cond	F (5, 70) = 7,387e-014	P > 0,9999
				Time	F (1, 70) = 20,97	P < 0,0001
**1F**	5-6	two-way ANOVA (Bonferroni)	Genotype/Conditions	Interaction	F (5, 58) = 4,797	P = 0,0010
			Time (time familiar location *vs* time novel location)	Gen/Cond	F (5, 58) = 1,476e-014	P > 0,9999
				Time	F (1, 58) = 17,58	P < 0,0001
**2B**	5-11	two-way ANOVA (Bonferroni)	Genotype (*Hdc* $^{+/+}$ *vs Hdc* $^{-/-}$)	Interaction	F (2, 100) = 1,537	P=0,2202
			Conditions (NS *vs* SCD *vs* SED)	Genotype	F (1, 100) = 0,08214	P=0,7750
				Conditions	F (2, 100) = 1,057	P=0,3515
**2D**	5-11	two-way ANOVA (Bonferroni)	Genotype (*Hdc* $^{+/+}$ *vs Hdc* $^{-/-}$)	Interaction	F (2, 38) = 3,655	P=0,0353
			Conditions (NS *vs* SCD *vs* SED)	Genotype	F (1, 38) = 9,536	P=0,0038
				Conditions	F (2, 38) = 9,732	P=0,0004
**2E**	6-9	two-way ANOVA (Bonferroni)	Genotype (*Hdc* $^{+/+}$ *vs Hdc* $^{-/-}$)	Interaction	F (2, 35) = 6,121	P = 0,0053
			Conditions (NS *vs* SCD *vs* SED)	Genotype	F (1, 35) = 0,1190	P = 0,7321
				Conditions	F (2, 35) = 1,203	P = 0,3123
**2F**	4-5	two-way ANOVA (Bonferroni)	Genotype (*Hdc* $^{+/+}$ *vs Hdc* $^{-/-}$)	Interaction	F (2, 22) = 6,648	P = 0,0055
			Conditions (NS *vs* SCD *vs* SED)	Genotype	F (1, 22) = 1,472	P = 0,2379
				Conditions	F (2, 22) = 4,010	P = 0,0327
**3A (Above)**	4-5	two-way ANOVA (Bonferroni)	Gen/Cond	Interaction	F (20, 105) = 2,027	P = 0,0114
			Fatty Acids	Gen/Cond	F (4, 105) = 66,33	P < 0,0001
				Fatty Acids	F (5, 105) = 0,6569	P = 0,6569
**3A (Below)**	4-5	two-way ANOVA (Bonferroni)	Gen/Cond	Interaction	F (40, 189) = 1,077	P = 0,3615
			Fatty Acids	Gen/Cond	F (8, 189) = 207,9	P < 0,0001
				Fatty Acids	F (5, 189) = 1,960	P = 0,0865
**3B**	4-5	two-way ANOVA (Bonferroni)	Gen/Cond	Interaction	F (2, 21) = 4,007	P = 0,0336
			Oxylipins	Gen/Cond	F (1, 21) = 3,781	P = 0,0654
				Oxylipins	F (2, 21) = 54,72	P < 0,0001
**3C**	4-5	two-way ANOVA (Bonferroni)	Gen/Cond	Interaction	F (2, 21) = 2,247	P = 0,1305
			Oxylipins	Gen/Cond	F (1, 21) = 2,036	P = 0,1683
				Oxylipins	F (2, 21) = 22,33	P < 0,0001
**3D**	4-5	two-way ANOVA (Bonferroni)	Gen/Cond	Interaction	F (2, 21) = 0,4135	P = 0,6666
			Oxylipins	Gen/Cond	F (1, 21) = 3,219	P = 0,0872
				Oxylipins	F (2, 21) = 1,637	P = 0,2184
**3E**	4-5	two-way ANOVA (Bonferroni)	Gen/Cond	Interaction	F (2, 21) = 0,1013	P = 0,9041
			Oxylipins	Gen/Cond	F (1, 21) = 0,1893	P = 0,6679
				Oxylipins	F (2, 21) = 2,902	P = 0,0771
**3F**	4-5	two-way ANOVA (Bonferroni)	Gen/Cond	Interaction	F (2, 21) = 3,170	P = 0,0626
			Oxylipins	Gen/Cond	F (1, 21) = 19,61	P = 0,0002
				Oxylipins	F (2, 21) = 3,931	P = 0,0355
**3G**	4-5	two-way ANOVA (Bonferroni)	Gen/Cond	Interaction	F (2, 21) = 3,470	P = 0,0499
			Oxylipins	Gen/Cond	F (1, 21) = 2,256	P = 0,1480
				Oxylipins	F (2, 21) = 2,860	P = 0,0797
**3H**	4-5	two-way ANOVA (Bonferroni)	Gen/Cond	Interaction	F (2, 21) = 1,444	P = 0,2586
			Oxylipins	Gen/Cond	F (1, 21) = 22,91	P < 0,0001
				Oxylipins	F (2, 21) = 3,823	P = 0,0384
**3I**	4-5	two-way ANOVA (Bonferroni)	Gen/Cond	Interaction	F (2, 21) = 2,027	P = 0,1567
			Oxylipins	Gen/Cond	F (1, 21) = 13,15	P = 0,0016
				Oxylipins	F (2, 21) = 2,385	P = 0,1166
**3J**	4-5	two-way ANOVA (Bonferroni)	Gen/Cond	Interaction	F (2, 21) = 1,421	P = 0,2637
			Oxylipins	Gen/Cond	F (1, 21) = 8,644	P = 0,0078
				Oxylipins	F (2, 21) = 3,173	P = 0,0625

Table S5. Statistical analysis of data shown in Figures S1–S2.

Figure	"n" (per group)	Analysis (post hoc reported in figures)	Factors Analyzed	F ratios		P values
**S1A**	27-34	two-way ANOVA (Bonferroni)	Genotype *($Hdc^{+/+}$ vs $Hdc^{-/-}$)*	Interaction	F (2, 179) = 2,573	P = 0,0792
			Conditions (NS *vs* SCD *vs* SED)	Genotype	F (1, 179) = 0,0003412	P = 0,9853
				Conditions	F (2, 179) = 1,250	P = 0,2889
**S1B**	12-16	two-way ANOVA (Bonferroni)	Genotype *($Hdc^{+/+}$ vs $Hdc^{-/-}$)*	Interaction	F (2, 76) = 0,4731	P = 0,6249
			Conditions (NS *vs* SCD *vs* SED)	Genotype	F (1, 76) = 0,01577	P = 0,9004
				Conditions	F (2, 76) = 0,01667	P = 0,9835
**S1C Central**	12-16	two-way ANOVA (Bonferroni)	Genotype *($Hdc^{+/+}$ vs $Hdc^{-/-}$)*	Interaction	F (2, 76) = 0,3171	P = 0,7292
			Conditions (NS vs SCD vs SED)	Genotype	F (1, 76) = 0,002588	P = 0,9596
				Conditions	F (2, 76) = 0,2941	P = 0,7460
**S1C Peripheral**	12-16	two-way ANOVA (Bonferroni)	Genotype *($Hdc^{+/+}$ vs $Hdc^{-/-}$)*	Interaction	F (2, 76) = 0,4802	P = 0,6205
			Conditions (NS vs SCD *vs* SED)	Genotype	F (1, 76) = 0,3501	P = 0,5558
				Conditions	F (2, 76) = 0,4165	P = 0,6609
**S1D Central**	12-16	two-way ANOVA (Bonferroni)	Genotype *($Hdc^{+/+}$ vs $Hdc^{-/-}$)*	Interaction	F (2, 76) = 1,158	P = 0,3197
			Conditions (NS vs SCD *vs* SED)	Genotype	F (1, 76) = 1,353	P = 0,2485
				Conditions	F (2, 76) = 2,776	P = 0,0686
**S1D Peripheral**	12-16	two-way ANOVA (Bonferroni)	Genotype *($Hdc^{+/+}$ vs $Hdc^{-/-}$)*	Interaction	F (2, 76) = 2,065	P = 0,1339
			Conditions (NS vs SCD vs SED)	Genotype	F (1, 76) = 0,2534	P = 0,6161
				Conditions	F (2, 76) = 1,122	P = 0,3308
**S1E Central**	12-16	two-way ANOVA (Bonferroni)	Genotype *($Hdc^{+/+}$ vs $Hdc^{-/-}$)*	Interaction	F (2, 76) = 0,6784	P = 0,5105
			Conditions (NS vs SCD vs SED)	Genotype	F (1, 76) = 1,348	P = 0,2492
				Conditions	F (2, 76) = 0,07071	P = 0,9318
**S1E Peripheral**	12-16	two-way ANOVA (Bonferroni)	Genotype *($Hdc^{+/+}$ vs $Hdc^{-/-}$)*	Interaction	F (2, 76) = 1,367	P = 0,2610
			Conditions (NS vs SCD vs SED)	Genotype	F (1, 76) = 0,02911	P = 0,8650
				Conditions	F (2, 76) = 1,366	P = 0,2613
**S2A**	5	two-way ANOVA (Bonferroni)	Genotype *($Hdc^{+/+}$ vs $Hdc^{-/-}$)*	Interaction	F (2, 24) = 1,120	P = 0,3427
			Conditions (NS vs SCD *vs* SED)	Genotype	F (1, 24) = 0,9532	P = 0,3386
				Conditions	F (2, 24) = 0,4840	P = 0,6222
**S2B**	5	two-way ANOVA (Bonferroni)	Genotype *($Hdc^{+/+}$ vs $Hdc^{-/-}$)*	Interaction	F (2, 24) = 2,600	P = 0,0950
			Conditions (NS vs SCD *vs* SED)	Genotype	F (1, 24) = 0,5018	P = 0,4855
				Conditions	F (2, 24) = 0,7303	P = 0,6222
**S2C**	4-5	two-way ANOVA (Bonferroni)	Genotype *($Hdc^{+/+}$ vs $Hdc^{-/-}$)*	Interaction	F (2, 23) = 1,988	P = 0,1599
			Conditions (NS *vs* SCD *vs* SED)	Genotype	F (1, 23) = 0,5971	P = 0,4476
				Conditions	F (2, 23) = 1,519	P = 0,2401
**S2D**	4-5	two-way ANOVA (Bonferroni)	Genotype *($Hdc^{+/+}$ vs $Hdc^{-/-}$)*	Interaction	F (2, 24) = 1,785	P = 0,1894
			Conditions (NS *vs* SCD *vs* SED)	Genotype	F (1, 24) = 0,1961	P = 0,6618
				Conditions	F (2, 24) = 3,537	P = 0,0451
**S2E**	4-5	two-way ANOVA (Bonferroni)	Genotype *($Hdc^{+/+}$ vs $Hdc^{-/-}$)*	Interaction	F (2, 22) = 1,776	P = 0,1927
			Conditions (NS *vs* SCD *vs* SED)	Genotype	F (1, 22) = 0,2656	P = 0,6114
				Conditions	F (2, 22) = 0,5681	P = 0,5747
**S2F**	4-5	two-way ANOVA (Bonferroni)	Genotype *($Hdc^{+/+}$ vs $Hdc^{-/-}$)*	Interaction	F (2, 21) = 1,477	P = 0,2511
			Conditions (NS *vs* SCD *vs* SED)	Genotype	F (1, 21) = 1,719	P = 0,2039
				Conditions	F (2, 21) = 0,4782	P = 0,6265
**S2G**	4-5	two-way ANOVA (Bonferroni)	Genotype *($Hdc^{+/+}$ vs $Hdc^{-/-}$)*	Interaction	F (2, 22) = 2,306	P = 0,1233
			Conditions (NS *vs* SCD *vs* SED)	Genotype	F (1, 22) = 1,209	P = 0,2834
				Conditions	F (2, 22) = 0,9529	P = 0,4010
